# Neutrophil Extracellular Traps in Cerebral Ischemia/Reperfusion Injury: Friend and Foe

**DOI:** 10.2174/1570159X21666230308090351

**Published:** 2023-08-15

**Authors:** Haoyue Luo, Hanjing Guo, Yue Zhou, Rui Fang, Wenli Zhang, Zhigang Mei

**Affiliations:** 1 Key Laboratory of Hunan Province for Integrated Traditional Chinese and Western Medicine on Prevention and Treatment of Cardio-Cerebral Diseases, College of Integrated Traditional Chinese Medicine and Western Medicine, Hunan University of Chinese Medicine, Changsha, Hunan, 410208, China;; 2 School of Pharmacy, Hunan University of Chinese Medicine, Changsha, Hunan, 410208, China;; 3 Third-Grade Pharmacological Laboratory on Chinese Medicine Approved by State Administration of Traditional Chinese Medicine, Medical College of China Three Gorges University, Yichang, Hubei, 443002, China

**Keywords:** Ischemic stroke, Alzheimer’s disease, cerebral ischemia, cerebral ischemia-reperfusion, neutrophil extracellular traps, neutrophil extracellular traps, CNS diseases

## Abstract

Cerebral ischemic injury, one of the leading causes of morbidity and mortality worldwide, triggers various central nervous system (CNS) diseases, including acute ischemic stroke (AIS) and chronic ischemia-induced Alzheimer's disease (AD). Currently, targeted therapies are urgently needed to address neurological disorders caused by cerebral ischemia/reperfusion injury (CI/RI), and the emergence of neutrophil extracellular traps (NETs) may be able to relieve the pressure. Neutrophils are precursors to brain injury following ischemic stroke and exert complicated functions. NETs extracellularly release reticular complexes of neutrophils, *i.e*., double-stranded DNA (dsDNA), histones, and granulins. Paradoxically, NETs play a dual role, friend and foe, under different conditions, for example, physiological circumstances, infection, neurodegeneration, and ischemia/reperfusion. Increasing evidence indicates that NETs exert anti-inflammatory effects by degrading cytokines and chemokines through protease at a relatively stable and moderate level under physiological conditions, while excessive amounts of NETs release (NETosis) irritated by CI/RI exacerbate the inflammatory response and aggravate thrombosis, disrupt the blood-brain barrier (BBB), and initiates sequential neuron injury and tissue damage. This review provides a comprehensive overview of the machinery of NETs formation and the role of an abnormal cascade of NETs in CI/RI, as well as other ischemia-induced neurological diseases. Herein, we highlight the potential of NETs as a therapeutic target against ischemic stroke that may inspire translational research and innovative clinical approaches.

## 1 INTRODUCTION

Cerebral ischemia, the most commonly reported leading cause of morbidity and mortality worldwide, is a major contributor to various central nervous system (CNS) diseases, such as acute ischemic stroke (AIS) and Alzheimer's disease (AD) [1-3]. Timely reperfusion treatment is the cornerstone of ischemic stroke care. Pharmacological thrombolysis and/or mechanical thrombectomy are available methods for ischemic penumbra to achieve recanalization and reperfusion [4]. However, the reperfusion caused by the sudden blood flow recovery often triggers cerebral ischemic reperfusion injury (CIRI). Although oxygen levels are restored upon reperfusion, pro-inflammatory neutrophils infiltrate the cerebral ischemic tissues to aggravate ischemic injury [5]. Neutrophils, one of the first cells to respond to cerebral ischemic injury, produce several inflammatory factors that aggravate brain-blood barrier (BBB) breakdown and cell death and block brain recovery [6]. Recently, the formation of neutrophil extracellular traps (NETs) has been regarded as a new defense mechanism [7, 8]. NETs are reticular complexes consisting of double-stranded DNA (dsDNA), histones, and granular proteins released by activated neutrophils in response to various stimuli [9]. The program of activation and formation of NETs comes to be known as NETosis [10]. Initially, NETs are described as a means for neutrophils to neutralize pathogens [7]. However, evidence has shown that NETs may contribute to cerebral ischemia/reperfusion injury [11]. NETs promote thrombosis by activating the coagulation cascade and a scaffold for platelet and red blood cell adhesion [12, 13]. Although NETs can reduce inflammation by hydrolyzing cytokines and chemokines [14] and help protect the host against pathogens [15], the components of NETs are nonspecific. In addition to controlling microorganisms, they induce neighboring tissue injured by firing the pro-inflammatory response [9]. NETs also carry proteins (*e.g*., myeloperoxidase (MPO), neutrophil elastase (NE), matrix metalloproteinases (MMPs), and histones) that directly impair the BBB [16]. Extravasated neutrophils release NETs within perivascular spaces and brain parenchyma [16]. NETs may be involved in neuronal death and microglia activation, further enhancing NETs release [17, 18]. Increasing evidence has demonstrated that strategies targeting the modulation of NETs may alleviate the damage caused by cerebral ischemia/reperfusion. The following article provides an overview of NETs formation and NETs in CI/RI, as well as the potential of NETs as therapeutic targets in ischemia-induced neurological disorders, such as AIS and chronic ischemia-induced AD.

## 2 METHOD

We searched PubMed, Web of Science, Google Scholar, and China National Knowledge Infrastructure (CNKI). Keywords included “neutrophil” or “neutrophil extracellular traps”, “cerebral ischemia” or “cerebral ischemia-reperfusion” or “ischemic stroke” or “Alzheimer's disease”. The searches identified English language papers and Chinese language papers published from the database establishment up to the present time. After carefully evaluating, we provide a comprehensive overview of the machinery about NETs formation and the role of NETs in CI/RI, as well as other ischemia-related neurological diseases, and discuss potential avenues for therapeutic intervention.

## 3 FORMATION OF NETS

Neutrophils are the most abundant circulating innate immune cells and form the first line of defense against invading pathogens and tissue injury [19, 20]. NETs are reticular complexes released extracellularly by activated neutrophils and consist of dsDNA decorated with histones and granular proteins, *e.g*., cathepsin G, MPO, NE, matrix metalloproteinase 9 (MMP-9) [7, 21]. Citrullinated histone 3 (CitH3) as a marker of NETs has been used to detect the existence of NETs in plasma and thrombus [22]. Furthermore, the elevated level of circulating CitH3 is positively associated with the severity and mortality of stroke [22, 23].

NETs can be initiated by recognizing several stimuli by neutrophil receptors such as toll-like receptors (TLRs) [24, 25]. Then, calcium ions stored in the endoplasmic reticulum are released into the cytoplasm, causing increased protein kinase C (PKC) activity, which induces NADPH oxidase (Nox) to produce reactive oxygen species (ROS) [24], which further continues to induce the activation of peptidyl arginine deiminase 4 (PAD4) [26]. PAD4 is essential for histone citrullination, a crucial step in forming NETs [11, 27]. PAD4 citrullinates the arginine residues of histones on chromatin, which causes chromatin decondensation and DNA naked [11]. NE was also considered to synergize with MPO, destroy chromosome integrity, and induce its disintegration [28]. Then the cell membrane breaks, and extracellular traps composed of DNA and proteins are released into the extracellular space [13]. This process of neutrophil death that depends on ROS production by Nox is called NETosis, which usually takes 3-4 h [12, 29]. In this case, blocking Nox suppresses ROS production and NETs release [29]. However, evidence indicates that NETs can be generated in the Nox-independent pathway without NETosis, which requires 5**-**60 mins [24]. Neutrophils extrude their decondensed chromatin with intact nuclear and cellular membranes, and cells retain their activity and phagocytic capacity [30]. Further studies have illustrated that the Nox-independent NETs formation process is facilitated through calcium influx and mitochondrial ROS [31]. On the whole, the Nox-dependent and Nox-independent pathways are two distinct pathways that lead to NETs formation. The activators and subsequently activated kinases needed for each pathway are different [11].

Additionally, recently emerging research studies have revealed that NETs may be associated with risk factors of ischemic stroke, such as the behavior of cigarette consumption, and medical comorbidities like atrial fibrillation (AF) and hyperglycemia. High *in vitro* glucose and hyperglycemia in patients have been shown to increase the release of NETs [32, 33]. An *in vitro* study has indicated that the common hypoglycemic drug metformin reduces the formation of NETs [34], and the potential mechanism is probably related to the inhibition of Nox activation [35]. A reduction in NETs markers was observed, primarily in CitH3 levels in patients with antecedents of smoking, while the clinical implication of this finding is currently unknown [23]. Additionally, NETs were increased in patients with AF history in the follow-up for 12 months after the ischemic event [23]. Notably, NETs may contribute to the pathogenesis of systemic lupus erythematosus [36], rheumatoid arthritis [37], atherosclerosis [38, 39], systemic vasculitides [40], as well as CNS diseases, including cerebral ischemic stroke [2], AD [41].

## 4 NETS IN CEREBRAL ISCHEMIA OR/AND REPERFUSION INJURY

**Fig. (1):**
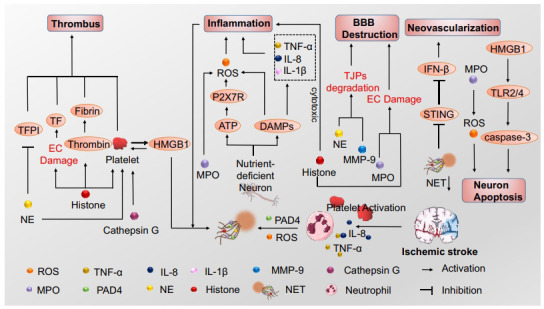
NETs formation and function after ischemic stroke. Neutrophils release neutrophil extracellular traps (NETs), extracellular reticular complexes that consist of double-stranded DNA (dsDNA), and a range of proteins in response to stimuli, such as platelet activation, interleukin-8 (IL-8) and tumor necrosis factor-α (TNF-α) after ischemic stroke. Reactive oxygen species (ROS) and protein arginine deiminase 4 (PAD4) are essential for NETs formation. NETs could promote thrombus formation through different mechanisms, including platelet activation, coagulation stimulation, and constituting a scaffold for platelets to adhere. NETs are also involved in blood-brain barrier (BBB) destruction, inflammation and neuron apoptosis. And inhibition of NETs formation promotes neovascularization.

In cerebral ischemic injury, neutrophils are activated through the action of released chemokines and cytokines and subsequently release NETs, which are involved in thrombosis, inflammation, destruction of the BBB, and neuronal damage (Fig. **1**). Recanalization of blood flow can salvage ischemic tissue, but sometime reperfusion causes tissue injury. The vital role of NETs in the above process has been supported by previous studies [42, 43].

### 4.1 NETs and Thrombosis during Cerebral Ischemia/Reperfusion Injury

Blocking blood flow by an occlusive thrombus leads to irreversible damage to associated brain tissue. Therefore, rapid removal of occlusive thrombus and establishment of early revascularization can effectively improve patient prognosis [44]. NETs form essential components of cerebral thrombus and have been experimentally demonstrated to promote thrombus formation [45], probably by activating the coagulation cascade. NETs exert a potent cytotoxic effect on endothelial cells by inducing phosphatidylserine exposure and tissue factor (TF) expression [46], directly inducing endothelial cell damage [47, 48]. The platelet adheres to the injury site and stimulates thrombus formation. Histones may damage endothelial cells and induce TF expression in vascular endothelial cells [49], activating coagulation *via* the extrinsic pathway [13]. Negatively charged DNA in NETs activates the endogenous coagulation pathway by directly providing a scaffold for the activation of coagulation factor XII [50]. Hydrogen peroxide (H_2_O_2_), a vital cause of NETs formation, may be released during histone-induced endothelial cell damage or even death [51]. Histones activate platelets through toll-like receptor 2 (TLR2) and TLR4 and enhance platelet aggregation by recruiting fibrinogen and inducing calcium influx [13, 52, 53]. Meanwhile, TLR4 removal considerably reduced the content of NETs in the ischemic brain [54]. Furthermore, by calcium influx, histones directly activate the alphaIIbbeta3 (αIIbβ3) integrin on platelet surfaces to induce platelet aggregation [53]. Additionally, NE and cathepsin G activates platelets through protease-activated receptors [55, 56]. It has been demonstrated that activated platelets could initiate and accelerate neutrophil-mediated externalization of chromatin in blood vessels [57]. Moreover, activated platelets present (high mobility group box-1 protein (HMGB1)) to neutrophils and commit them to the generation of NETs. Once NETs are generated, HMGB1, in turn, activates platelets and promotes their recruitment and activation by presenting histones to platelets TLR2 and TLR4 [52, 58, 59]. NETs components have been shown to increase thrombus production by regulating or degrading endogenous anticoagulants. For example, NE inactivates tissue factor pathway inhibitors (TFPI), thus resulting in increased procoagulant activity [60]. These circumstances may be blamed for exacerbating cerebral ischemia and other diseases associated with immune thrombosis. NETs provide a scaffold for platelet and red blood cell adhesion and improve the stability of blood clots [12]. The researchers perfused NETs with platelets suspended in plasma. They observed that small platelet aggregations appeared on NETs within 1 min from the onset of perfusion, and platelet adhesion and aggregation on NETs increased in the next 9 min. Additionally, the attachment of individual red blood cells to NETs has been observed using electron microscopy [12]. The study has revealed that the reperfusion rate after tissue-type plasminogen activator (t-PA) administration was low even though the treatment was started within hours of symptom onset [57, 61], suggesting that the thrombus contained a scaffold component independent of fibrin. DNase 1 has been identified as a key driver of NETs degradation [62]. Digestion of NETs with DNase and fibrin with t-PA showed that t-PA removed fibrin but did not prevent clot formation, and the use of both DNase and t-PA was effective in preventing clot formation, suggesting that NETs provide a scaffold independent of fibrin and t-PA with no significant effect in digesting this scaffold [12]. Additionally, NETs activate platelets and endothelial cells, stimulating a procoagulant phenotype and facilitating von Willebrand factor (vWF) and plasminogen activator inhibitor-1 (PAI-1) release, thereby reversing the fibrinolytic effects [63].

NETs induce thrombus formation in cerebral ischemia through several mechanisms, and thrombus NETs content may be responsible for reperfusion resistance [64]. However, increased circulating NETs after thrombolytic therapy is associated with increased disease severity [63]. Recent research has discussed the impact of individual NETs components, such as DNA, NE, and histone, on coagulation. However, the mechanism of how complete NETs promote thrombus formation is still to be investigated [57].

### 4.2 NETs and Inflammatory Response

Under physiological conditions, NETs immobilize pathogens and restrict their spread, thus possessing a critical antimicrobial function within the innate immune system [65]. NETs resolve inflammation by proteolysis of cytokines and chemokines and protection from antiproteases [14]. However, NETs would exacerbate inflammation under conditions of excess or clear insufficiently. In cerebral ischemia/reperfusion, the deleterious impact of NETs is more pronounced and deserves our attention.

In cerebral ischemia, nutrient-deficient neuronal cells rapidly release damage-associated molecular patterns (DAMPs), which mediate pro-inflammatory activation signals of microglia, astrocytes, and endothelial cells [59, 66]. Activated platelets release DAMPs, further stimulating inflammation in cerebral ischemic injury [67]. A critical DAMP that platelets release is HMGB1, a DNA-binding protein with procoagulant and pro-inflammatory functions. The latest study suggests that platelet-derived HMGB1 mediates the formation of NETs in stroke [68]. The inflammatory response is exacerbated by HMGB1, which activates TLR2/4 on the surface of neutrophils, causing the assembly of Nox and ROS production and increasing the production of the antiapoptotic protein Bcl-xl and pro-inflammatory cytokines [26, 69]. Moreover, HMBG1 levels can predict reperfusion injury in patients with ischemic stroke [70]. It is vital to mention that adenosine triphosphate (ATP), like HMGB1 [18], is also a component of the cellular content extruded during NETosis [71]. Extruded ATP may aggravate the inflammatory response in the ischemic brain by further recruiting and activating surrounding neutrophils and other immune cells [71]. As a result, a vicious cycle is generated, driven by ATP, that exacerbates the inflammatory response after permanent middle cerebral artery occlusion (MCAO). It is worth mentioning that histones and granulins in the fibrous meshwork of NETs are cytotoxic and can induce acute inflammatory responses [42, 72]. MPO may mediate oxidative injury and inflammation by producing Hypochlorous acid (HOCl) [73]. MPO activation triggers the production of HOCl by catalyzing the interaction between chloride and H_2_O_2_ [74-76]. HOCl shows great diffusivity and oxidative activity that react with lipids, proteins, DNA, and itself, which can exacerbate oxidative stress and mediate the generation of superoxide, peroxynitrite, and oxidized endothelial NO synthase (eNOS) dimers in endothelial cells [77-82]. In an experimental ischemic stroke animal model with postponed t-PA administration, therapy with taurine, a HOCl scavenger, decreased the rates of hemorrhagic transformation [83].

### 4.3 NETs and BBB Destruction

As the gatekeeper of the CNS, the BBB is a functional interface that separates the brain from the circulatory system and is important for CNS homeostasis [84, 85]. Under physiological conditions, there are few neutrophils in the CNS because of BBB. Meanwhile, under pathological conditions, neutrophils are one of the first blood-derived cells to be attracted to ischemic tissue after cerebral ischemia [86]. In a mouse model of acute cerebral ischemia, neutrophil infiltration of the capillary lumen, perivascular space, and brain parenchyma with NETs formation features such as CitH3 positivity, chromatin decondensation, and extracellular projection of DNA and histones have been found [87].

MMPs have been confirmed as crucial mediators of BBB destruction following cerebral ischemia and reperfusion [85]. MMPs degrade almost all extracellular membrane proteins, causing BBB hyperpermeability and disruption of barrier integrity [88]. MMP-9, one of the most widely investigated MMPs, directly degrades extracellular matrix proteins [89, 90]. Tight junctions present between the cerebral endothelial cells form a diffusion barrier, which selectively excludes most blood-borne substances from entering the brain [91]. Studies have informed that MMP-9 leads to BBB destruction by causing degradation of the tight junctional and extracellular matrix, further contributing to neuronal death [92, 93]. t-PA thrombolysis activates MMP-9 and other molecular pathways that may result in acute BBB disruption [94]. It is important to note that, despite mediating deleterious effects in the acute phase of stroke, MMPs may play a beneficial role in the delayed recovery phase after stroke [26]. Upon blood flow recanalization, MMPs released at low levels could reshape the basement membrane and reinstate cell-to-cell contacts, thus enabling vasculogenesis and BBB reestablishment [94].

Additionally, other enzymes in NETs, such as NE, MPO, and histones, can cause BBB leakage. PAD4 overexpression results in magnified vascular injury and BBB destruction and reduces neovascularization by releasing more NETs [95]. NE increases endothelial permeability and intercellular cell adhesion molecule-1 (ICAM-1) expression on endothelial cells, thus can damage the BBB [96]. MPO and histones are rich proteins in NETs and induce endothelial cell death and BBB disruption [97].

### 4.4 NETs and Neuronal Death

Neutrophils are attracted to the ischemic tissue after stroke and activated by tumor necrosis factor-α (TNF-α), interleukin-1β (IL-1β), and interleukin-8 (IL-8) secreted by microglia or astrocytes, followed by the release of NETs, which may inversely activate glial cells and lead to neuronal damage [11]. Polarization of neutrophils toward the N2 phenotype has been shown to have neuroprotective effects in ischemic stroke, while *in vitro* data demonstrated that neuronal ischemia drove neutrophils away from the N2 phenotype and exacerbating the formation of NETs, further increasing neuronal death after ischemic injury [98, 99].

Previous studies have illustrated that the accumulation of ATP in ischemic brain tissue probably mediates the circulation between the formation of NETs and neuronal injury and may exacerbate inflammation and brain injury [71]. Updated studies suggest that HMGB1 is passively released from necrotic cells and then binds to its target receptors, such as TLR2, TLR4, and the receptor for advanced glycation end products (RAGE), stimulating the expression and release of additional pro-inflammatory mediators through the positive feedback loop of nuclear factor-κB (NF-κB) signaling pathways, ultimately causing neuronal loss and apoptosis [100-102]. Moreover, TLR2 and TLR4 expression levels and ischemia-induced cleavage of apoptotic protease caspase-3 were significantly increased in the presence of HMGB1 [103]. HMGB1 promotes the production of NETs and recruits immune cells that exacerbate neuroinflammation, and it is a part of NETs and is involved in NETosis-mediated neuronal death [18]. Administration of anti-HMGB1 antibodies to the ischemic brain has been reported to inhibit NETs production, reduce neuronal death after stroke, and reduce subsequent neuroinflammation [100]. HMGB1 exerts a good predictive value for cerebral ischemia-reperfusion injury patients, and the increased expression is correlated with a worse prognosis [70]. HMGB1 is involved in astrocyte repair and reconstruction and stimulates neurovascular repair in the late stage of ischemic injury [100].

Additionally, proteases associated with NETs, such as MPO, affect post-stroke neurogenesis [73]. The MPO inhibitor N-acetyl lysyltyrosylcysteine amide (KYC) decreases M1 microglia and N1 phenotype neutrophils, improves neuronal stem cell proliferation and differentiation in the ischemic cortex, and safeguards exogenous neural stem cells in the ischemic brain [104]. However, more research is required because the direct mechanisms by which NETs affect nerve cells are currently not well studied.

## 5 NETS AND ISCHEMIA-INDUCED NEUROLOGICAL DISEASES

### 5.1 Targeting NETs to Regulate Ischemic Stroke

Ischemic stroke is a primary cause of death and disability worldwide, affecting millions of people each year to the point where AIS represents 87% of the overall prevalence of stroke [105, 106]. Intravenous thrombolysis and endovascular thrombectomy are the effective primary treatments for early ischemic stroke [107, 108], despite their limitations, such as many contraindications and short treatment windows [109, 110].

Neutrophils are precursors to brain lesions after ischemic stroke and exert elaborate functions [99]. One of the functional traits of neutrophils is the creation of extracellular traps. Research has shown that NETs exacerbate injury after transient MCAO (tMCAO) in mice by releasing transmigrated neutrophils in stroke lesions [17, 45]. Both the suppression of NETs formation and the strengthening of NETs lysis in the lesion are possible therapeutic targets for ischemic stroke and should be investigated in depth [67].

Targeting NETs reduces oxidative stress in stroke. Cerebral ischemia/reperfusion triggers various biochemical and cellular responses that produce excessive ROS [111]. Subsequent oxidative stress is one of the central pathophysiological processes in ischemic stroke [112, 113]. Under oxidative stress, endogenous redox is out of balance. ROS causes cytotoxicity through oxidative damage to such nucleic acids, lipids, and proteins, inflicting damage on brain tissue structures [114, 115]. Oxidative stress causes neuronal apoptosis, inflammatory signaling pathways activation, and BBB impairment, all of which promote neurodegeneration and cell death in ischemic stroke [116-119]. NETs formation is triggered by ROS, and the latest investigation reveals that mitochondrial ROS are adequate for producing them [120-122]. MPO and NE perhaps bridge the link between oxidative stress and NETs. MPO is not only a critical enzyme involved in the production of free radicals within the oxidative burst but also involved in chromatin depolymerization, an essential step in NETs [123]. After ROS generation, NE leaves the azurophilic granules and translocates to the nucleus, where MPO subsequently binds to chromatin, and the two enzymes cooperate to enhance chromatin depolymerization, causing cell rupture [124]. This mechanism could probably be a target in future studies. A recent study revealed that macrophage migration inhibitory factor (MIF) initiates HOCl production activity by phagocytic neutrophils and contributes to the production of antimicrobial hypochlorite acid-producing MPO, thus inhibiting the formation of NETs and suppressing oxidative stress [125]. The experimental results showed that cationic solid lipid nanoparticles (cSLNs) increased respiratory burst and degranulation through Ca^2+^ influx and the MAPK pathway [126, 127]. NETs were produced within minutes of this reaction, accompanied by a 24- and 9-fold increase in superoxide anion and elastase levels, respectively, although this phenomenon was insignificant for neutral SLNs (nSLNs) [126]. It is worth mentioning that avermectin (AVM) can inhibit the release of NETs and MPO expression and decrease respiratory burst levels by activating PTEN demethylation [128]. Additionally, hydrogen sulfide upregulated miR-16-5p targeting PIK3R1 and RAF1, which reduced the level of respiratory burst and thus inhibited the formation of NETs [129]. However, these studies in human health are immature.

Targeting NETs reduces local thrombosis and the inflammatory response in stroke. A fundamental process in the complicated pathophysiology of ischemic stroke is gradual thrombosis, leading to cerebrovascular occlusion [130]. Re-establishing recanalization of occluded blood vessels that supply oxygen and nutrients to the brain is the main therapeutic aim to limit ischemic brain injury [131]. However, CIRI involves both thrombotic and inflammatory pathways acting in concert to cause tissue damage [132]. Given the limited therapeutic strategies in stroke management, it is essential to develop new therapeutic approaches for ischemic stroke targeting thrombotic inflammation [133]. Currently, rich neutrophils and NETs are largely observed in thrombi carried by patients with ischemic stroke [45]. Platelets are known to play a role in NETs formation [134], while NETs have been observed primarily in platelet-rich areas of ischemic stroke thrombi and microthrombi in the brain [67]. As platelets interact with neutrophils, NETs formation is induced by different mechanisms, such as DAMPs, including HMGB1 [68, 135] and ATP [71]. HMGB1 is considered a candidate NETosis inducer in noninfectious diseases [136]. In a mouse model of ischemic stroke, platelets mediate HMGB1 release, resulting in detrimental NETs formation [67]. Conversely, platelet-specific HMGB1 knockouts block platelet-induced NETs formation and improve stroke outcomes [67]. BoxA, a competitive HMGB1 inhibitor, also achieves a marked reduction in platelet-induced NETs formation [135]. Together, the above findings confirm the critical role of platelet-derived HMGB1 in forming detrimental NETs in the acute phase of ischemic stroke. The study illustrates that NETosis involving HMGB1 as a mediator in a vicious cycle aggravates inflammation and subsequent damage in the ischemic brain [18]. Targeting NETosis by modulating HMGB1 may provide a versatile therapeutic strategy to mitigate ischemic brain damage. Furthermore, in the current study, ATP is a DAMP molecule that accumulates in the brain and induces NETs in the brain parenchyma and in circulating neutrophils [71]. ATP indirectly induces NETs through P2X7R-mediated activation of the NLRP3 inflammasome, causing cell death and NET release [137, 138]. A recent study has revealed that ATP significantly enhances the induction of PAD4 and CitH3 and intracellular Ca^2+^ influx, PKCα activation, and Nox-dependent ROS production in a P2X7R-dependent manner [71]. In MCAO animal models, NETosis was significantly inhibited by treatment with apyrase, an enzyme that hydrolyzes ATP but enhanced by co-treatment of BzATP, confirming ATP-P2X7R-mediated NETosis [71]. Panx1, which forms ATP channels in neutrophils, likely contributes to ATP release during NETosis, especially in inflammation [139-141]. Importantly, pharmacological inhibition of Panx1 channels with BB-FCF (an FDA-approved blue dye for food coloring) is sufficient to minimize the number of neutrophils undergoing NETosis due to A23187 and PMA [141]. Platelet TLR4 has been found to play a critical role in stroke injury in a NET-dependent manner [54], and upregulation of TLR4 is associated with higher inflammation and poorer outcomes in stroke [142, 143]. This finding provides a new avenue for treating AIS by inhibiting or degrading NETs. A recent study found that the agaphelin elastase inhibitor protects against AIS in mice by reducing thrombosis, BBB damage, and inflammation [130]. The antihematogenic and anti-inflammatory effects of agaphelin *in vitro* and *in vivo* are attributed to its inhibition of the catalytic activity of NE [144]. Previous studies have shown that NE contributes to the reduction of cathepsin G-induced platelet aggregation, TFPI cleavage, degradation of MMP-9, attenuated neutrophil chemotaxis, and decreased release of NETs [120, 145, 146]. Hence, elastase inhibition is potentially associated with the blockade of several relevant pathways contributing to thrombosis and neutrophil-mediated inflammation [130, 144]. Additionally, neutrophil-derived MPO activation has been proposed to be associated with inflammation and the magnitude of brain injury during the period of ischemic stroke [73]. 4-aminobenzoic acid hydrazide (ABAH), an irreversible specific MPO inhibitor, has been found to improve multiple sclerosis (MS) and stroke in experimental models [147, 148]. Heat shock proteins (Hsps) have multiple functions, including neuroprotective, anti-apoptotic, and anti-inflammatory effects in stroke [149, 150]. Post-insult ABAH treatment increased cytoprotective protein levels of cytoprotective proteins (*e.g*., Hsp70) but decreased pro-apoptotic p53 in the ischemic brain and promoted cell survival to improve functional outcomes [151]. In summary, MPO inhibition is a potentially promising therapeutic strategy for stroke. Notably, targeting the NET-VWF signaling pathway is suggested as a potential treatment strategy for AIS [152]. All of the above may provide a new route for the future treatment of ischemic stroke.

Post-stroke depression (PSD) is the most common acute psychiatric complication of ischemic stroke and has become a major barrier to stroke recovery [153]. The mechanisms underlying PSD are complex and have received little attention in the field of neurobiology. However, there is still evidence that neuroinflammation may be involved in PSD processes [154, 155]. Excessive release of NETs facilitates neuroinflammation by releasing metalloproteinases, proteases, cytokines, extracellular histones, DNA, and ROS [47]. Interleukin (IL)-17 and its congeners as mediators of tissue damage in the delayed phase of the inflammatory cascade response [156], and IL-17 production is suggested to recruit neutrophils to tissue and amplify systemic inflammation [157]. NETs have demonstrated the ability to activate inflammatory Th17 cells, which secrete IL-17, in diseases related to experimental autoimmune encephalomyelitis (EAE) [11], multiple sclerosis (MS) [158], and myocardial infarction (MI) [159, 160]. Additionally, relevant clinical research has confirmed that IL-17 is a significant player in PSD development by promoting the inflammatory response [161]. Considering the role of neutrophils and NETs in neuroinflammation in stroke and the pathogenesis of PSD, we suspect that NETs may be involved in post-stroke depression.

Targeting NETs improves vascular remodeling during stroke recovery. Neovascularization and perfusion of vascular structures around the ischemic cerebral play an essential role in recovery from cerebral ischemia. Restoring blood flow and oxygen supply to the ischemic tissue is fundamental for ischemic brain repair [162]. NETs formation is detected in blood vessels, and cerebral parenchyma in the periphery cortical area of infarction impairs revascularization and vascular remodeling after cerebral ischemia [95, 163]. Intravenous t-PA thrombolysis is the only FDA-approved treatment for patients with AIS. Treatment with intravenous t-PA within 3 h of ischemic stroke onset improves clinical outcomes at three months but triggers an increased incidence of symptomatic intracerebral hemorrhage [164]. It has been shown that t-PA directly stimulates neutrophils from ischemic mice to release NETs *via* upregulation of LRP-1 and PAD4 [165]. PAD4 forms a complex with calcium and rapidly translocates into the nucleus [166, 167, 168], and catalyzes the conversion of positively charged arginine on histone H3 to neutral guanine [168-170], which leads to the disruption of ionic interactions (*e.g*., hydrogen bonds) in chromatin and results in histone depyrogenation. Gene expression begins when chromatin changes to a relaxed state [168]. In summary, PAD4 has a regulatory role in NETs formation by mediating chromatin decondensation through histone citrullination [171]. However, overexpression of PAD4 exacerbates BBB destruction and reduces revascularization [57]. PAD4 deficiency or pharmacological inhibition increases neovascularization and vascular repair and improves functional recovery [95]. Data revealed that PAD inhibitor Cl-amidine administration inhibited STING pathway activation and interferon β (IFN-β) production [95]. Stroke leads to upregulation of the DNA sensor STING, activates IFN regulatory factor (IRF3) and TANK-binding kinase 1 (TBK1), and induces IFN-β synthesis1. Silencing STING or administering blocking antibodies to the IFN receptor to mice increased vascular regeneration and significantly reduced BBB damage. Thus, STING-mediated IFN responses are probably associated with NETs and ischemic vascular remodeling. Furthermore, administration of NETs digested with DNase 1 markedly decreased BBB destruction, while pericyte coverage on micro-vessels increased, and new functional vessels were formed, contributing to stroke recovery [95].

Notably, Icariside II (ICS II), a flavonoid derived from Herba Epimedii, has many beneficial activities, such as anti-osteoporotic, anti-aging, and anti-inflammatory properties and effects against erectile dysfunction [172-175]. It has drawn increasing attention due to the discovery of its role in neurological diseases [176, 177]. The current study revealed that ICS II significantly ameliorates I/R-induced BBB disruption in MCAO mice by regulating the balance of MMP9/TIMP1 and further inhibiting neuronal apoptosis by a caspase 3-dependent apoptosis pathway, thereby protecting against CI/RI [175]. These findings indicate that NETs may be a vital target for treating cerebral ischemia-induced neovascularization injury and functional recovery (Table **1**) [176-179].

### 5.2 Targeting NETs to Regulate Chronic Ischemia-induced AD

AD is a gradual and progressive neurodegenerative disease [180], which is characterized by neurotic plaques of amyloid-β (Aβ) deposition and abnormal hyperphosphorylated tau protein aggregation in the soma of neurons [181]. Increasing evidence indicates that cigarette smoking, midlife high blood pressure, obesity, diabetes, and cerebrovascular lesions are potential risk roles in AD [182]. Additionally, studies have revealed that cerebral blood flow (CBF) is reduced, ranging from 10% to 28% in patients with AD and other neurodegenerative illnesses [183, 184].

**Fig. (2):**
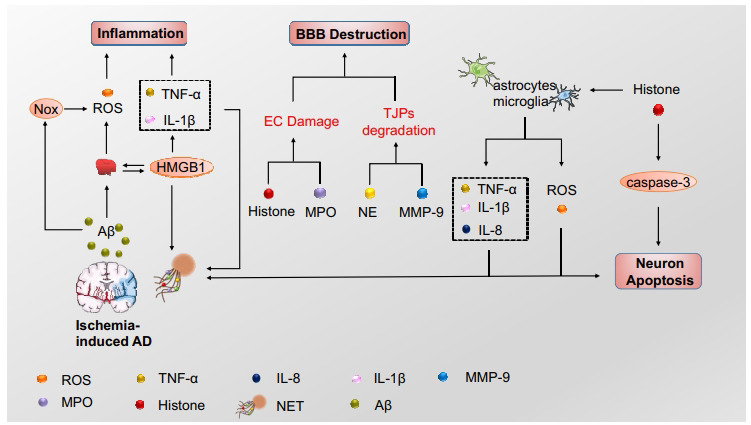
NETs in ischemia-induced Alzheimer's disease. In the AD brain, upregulated amyloid-β (Aβ) stimulates platelet activation to release HMGB1, which promotes NETs formation. Aβ, astrocytes, and microglia release ROS and proinflammatory cytokines, inducing NETs formation, which involves blood-brain barrier (BBB) destruction, inflammation, and neuron apoptosis.

Aβ may be involved in endothelial activation and intravascular neutrophils adhesion in AD. Researchers discovered that neutrophils exudate and are present in regions with Aβ deposits, where they released NETs [185]. Aβ peptides induce the expression of intercellular adhesion molecule-1 (ICAM-1) and circulate neutrophils to adhere to blood vessels by binding to ICAM-1 through lymphocyte function-associated antigen-1 (LFA-1) [96]. Blocking neutrophil depletion or inhibiting neutrophil transportation by LFA-1 reduced AD-like neuropathology and enhanced memory in mice already exhibiting cognitive dysfunction in AD models [185]. Metformin reduced neutrophil infiltration, thus lessening endothelial injury and lowering BBB permeability *via* down-regulate ICAM-1 in an AMPK-dependent manner [186]. Studies have illustrated that neutrophils that transiently attach to the endothelial cell walls of brain capillaries cause most CBF reduction, and the administration of anti-Ly6G antibody targeting the neutrophil-specific protein Ly6G disturbs neutrophils adhesion immediately and drives rapid improvements in CBF in AD mouse models and enhanced short-term memory function [184, 187]. Endovascular NETs are released by adhered neutrophils and may be related to pro-inflammatory cytokines or activated platelets. TNFα, IL-1β, and IL-8 are released at high levels by AD brain microvessels compared to non-AD microvessels [188, 189]. Furthermore, the previous study has revealed that TNFα, IL-1β, and IL-8 are involved in the enhanced release of NETs [190]. Platelets are preactivated in the blood of AD-transgenic mice, showing strong enhancement upon stimulation, and are considered a biomarker for early diagnosis of AD [191, 192]. Extensive evidence shows that platelets exposed to Aβ stimulate platelet activation and the generation of Aβ and ROS, initiating a vicious spiral that increases vascular inflammation [192-194]. Interaction between activated platelets and neutrophils through the TLR4 or LFA-1 integrin can induce intravascular NETs [185]. Activated platelets present HMGB1 to neutrophils, resulting in the production of NETs, and HMGB1 activates neutrophils, triggering the release of pro-inflammatory cytokines and further increasing inflammation in AD [59] (Fig. **2**). Blocking HMGB1 activity may provide a new therapeutic method for AD [194, 195]. RAGE is one of the receptors on the neutrophil surface, which may interact with HMGB1 inducing NETs formation in AD, and it has been shown that anti-RAGE antibody treatment could prevent NETs formation [135]. Oral pretreatment of glycyrrhizin, a small molecule inhibitor of extracellular HMGB1 cytokine activity, could inhibit HMGB1, thus reducing neuroinflammation and Alzheimer's-related pathology in the hippocampus of aged mice [195, 196]. Anti-HMGB1 mAb blocks HMGB1 activity with TLR4 and inhibits the HMGB1-induced elevation of Aβ monomers and oligomers [197]. NETs damage the endothelial wall by releasing NE, MMPs, MPO, and histones and further induce BBB destruction. MPO and histones in NETs can trigger endothelial cell death [97]. A previous study indicated that MPO-deficient AD mice had a better cognitive and lower inflammatory response than MPO-expressing AD mice [198]. MMP-9 and NE may disrupt tight junctions, thus promoting endothelial cell injury. NE increases endothelial permeability and ICAM-1 expression on endothelial cells, thereby damaging the BBB [199, 200]. Resveratrol may maintain the integrity of the BBB through the reduction of MMP-9 in transgenic mouse AD models [201]. Neutrophils enter the brain parenchyma at the early stage of AD, and intraparenchymal migrating neutrophils produce NETs [185]. In AD patients, activated astrocytes and microglia release pro-inflammatory cytokines and ROS into nearby brain tissue rich in Aβ deposits and may consequently assist in the production of NETs, which further activate glial cells and damage neighboring neurons [96]. Additionally, Aβ stimulates the production of ROS, which is essential for NETs formation by activating Nox in both human and mouse neutrophils *in vitro* [57, 96]. MMPs and NE are involved in the degradation of extracellular matrix proteins. NE and MPO also cause the degradation of tissues by activating MMPs and inactivating the endogenous tissue inhibitors of MMPs (TIMPs) [199, 202, 203]. Extracellular histone H1 has been identified in amyloid plaques. It triggers a pro-inflammatory response in microglia and leads to neuronal death by activating the mitochondrial apoptosis pathway in brains with AD (Table **2**) [204, 205].

## 6 CLINICAL APPLICATIONS AND PROSPECTS OF NETS

Administrating DNase and t-PA can effectively digest thrombus obtained from stroke patients [18]. Unfortunately, thrombolysis with t-PA has many limitations, such as the narrow therapeutic window and safety concerns about intracerebral hemorrhage and neurotoxicity [64, 208, 209]. The administration of DNase *in vitro* accelerates t-PA-induced thrombolysis [64] and does not exacerbate damage to BBB of MCAO mice [54]. These may indicate new approaches for recanalizing vessels with platelet-rich thrombus after stroke, especially to overcome t-PA resistance. DNase might be considered a medicine for AIS recanalization therapy for the ability to digest NETs. We expect DNase combined with fibrinolytic therapy to improve the prognosis of patients with ischemic stroke.

NETs and NETs markers may have diagnostic or prognostic marker values for patients with ischemic stroke. Research shows that the circulating level of NETs and dsDNA increased in patients with AIS in the initial phase, indicating the possibility that NETs may serve as a marker for the early diagnosis of ischemic stroke [210]. Additionally, the levels of NETs markers are associated with stroke severity. NETs markers (cfDNA, nucleosomes, and CitH3) were measured at onset and discharge in 243 patients with AIS and were found to be significantly high in the plasma of patients with AIS compared with healthy subjects and increased in patients over 65 years of age with a history of AF, myocardial infarction stroke, hyperglycemia, and severe stroke scores at admission and discharge [23]. Elevated levels of CitH3 at presentation were associated with AF and all-cause mortality in one year [23]; this indicates that CitH3 may be a useful prognostic marker and a potential therapeutic target for patients with ischemic stroke. However, more detailed research is required to accurately and quickly determine circulating levels of NETs in a clinical setting. Edaravone was first used in China and Japan as a free radical scavenger to treat AIS clinically [211, 212]. Edaravone Dexborneol (Eda.B), the combination of Edaravone and Borneol [213], received approval from the China National Medical Products Administration and was clinically used for AIS patients [214]. It has been proposed that the protective effect of Eda.B may be related to the ability to reduce NETs levels [214]. However, the particular mechanism of this new synthetic medicine is insufficiently understood. Neutrophil phagocytosis is essential in host defense, and any therapies targeting the formation of NETs must avoid impairing the physiological functions of these cells [29].

## CONCLUSION AND PERSPECTIVE

In summary, Nox-dependent and Nox-independent pathways are different ways of NETs formation [11]. The ability of NETs to trap microorganisms generated much enthusiasm initially, but their pathogenic potential in CI/RI has caught our attention. As stated above, NETs exacerbate the inflammatory response, aggravate thrombosis, disrupt the BBB, and initiate sequential neuronal injury and tissue damage. Therefore, inhibiting NETs formation or improving NETs resolution in disease may be a therapeutic target for ischemia-induced neurological disorders, such as AIS and AD, which are worthy of in-depth investigation. Notably, we speculate that NETs may also be involved in the pathogenesis of neurological complications after stroke.

Although the role of NETs in the pathogenesis of neurological diseases induced by ischemia has not been fully elucidated, it has attracted increasing attention, which will positively impact both improving the understanding of disease and refining therapeutic approaches in the future. A better understanding of the function and impact of NETs will allow us to suppress deleterious qualities without affecting positive ones and, eventually, enable us to exploit NETs to treat diseases.

Taken together, we aim to improve the outcomes of patients with ischemic stroke and ischemia-induced AD by targeting NETs. However, some limitations exist in the current studies. Firstly, owing to tissue processing techniques and other limitations, many studies have not been able to show robust extracellular NETs. And NETs are 3D lattices that are best imaged in their native state because of these limitations. Using intravital microscopy of the brain during ischemic injury to visualize the NETs formation in the future may refine research. Secondly, although the characteristic morphological changes in the shape of neutrophil nuclei can be used to detect NETs, they require sophisticated instrumentation and analytical methods. Citrullinated histones have originally been used as NET markers, but they are only specific to PAD4-mediated NETs formation. Thirdly, targeting specific steps or products of NETosis can offer therapeutic benefits in NET‐associated diseases. Multiple drugs targeting different steps of NETs formation have been reported, including Cl‐amidine, HDQ, DPI, NAC, rhDNase, vitamin D, antibiotics, and others. However, these drugs are associated with negative effects on the host's immune system, such as increased susceptibility to infections and weakened immune systems. Combined therapy might be an effective approach to reduce these detrimental effects and improve their efficacy. Further studies are recommended to reveal the detailed connections between NETosis and NETosis‐related diseases and identify strategies to modulate dysregulated NETosis effectively.

## Figures and Tables

**Table 1:** Possible strategies for targeting NETs in Ischemic stroke.

**Diseases**	**Strategies**	**Model**	**Effect and Function**	**References**
Ischemic stroke	Knockout platelet TLR4	TLR4^−/−^ mice pMCAO model	Inhibited NETs formation by downregulated TLR4	[54]
	Administration of DNase-I	Mice MCA electrocoagulation model	Disruption of NETs by digesting DNA	[54, 95]
		Mice pMCAO model		
	Platelet-specific knockdown of HMGB1	Mice tMCAO model	Reduced platelet-induced NETs formation	[67]
	nNIF therapy	Mice tMCAO model	Inhibited NETs formation by suppressing PAD4, neutrophil nuclear histone citrullination	[67, 178]
	Cl-amidine therapy	PAD4^−/−^ mice MCA electrocoagulation model	Inhibition of NETs release *via* suppressing STING pathway activation and IFN-β production	[95]
	Administration of 4-IPP	MIF knockout mice cell model	Inhibition of MIF increases neutrophil HOCL production capacity, thus showing a decrease in a NET release	[125]
	Applying cSLNs	Human cell model	Increased respiratory bursts and degranulation through Ca^2+^ influx and MAPK pathways, thereby promoting NETs production	[127]
	Administration of AVM	Carp cell model	Inhibited NETs release by activating PTEN demethylation	[128]
	Applying H_2_S	Vitro H_2_S exposure model for neutrophils	Inhibited NETs formation by upregulating miR-16-5p targeting PiK3R1 and RAF1	[129]
	Agaphelin therapy	Mice tMCAO model	Inhibited NE-dependent NET formation by inhibiting NE catalytic activity	[130]
	BoxA/anti-RAGE mAbs	Acute myocardial infarction patients	Reduced extracellular DNA released by neutrophils attacked by activated platelets or HMGB1	[135]
	BB-FCF therapy	Mice cell model	Inhibited NETs formation by inhibiting Panx1-ATP channel	[141]
	ABAH treatment	MPO^−/−^ mice tMCAO model	Suppressed MPO by increasing Hsp70 and p-Akt and weakening p53	[151]
	DNase I treatment or PAD4 deficiency	Mice MCAO tPA treatment model	Suppressed NET generation	[165]
	Administration of ICS II	Rat MCAO model	Regulated the balance of MMP9/TIMP1	[175]
	Administration of Apyrase	Rat pMCAO model	Inhibited NETs formation by hydrolysis of ATP	[179]

**Table 2:** Possible strategies for targeting NETs in ischemia-induced Alzheimer’s disease.

**Diseases**	**Strategies**	**Model**	**Effect and Function**	**References**
Ischemia-induced Alzheimer’s disease	Using Ly6G antibody	APP/PS1 mice AD model	Reduced neutrophil adhesion and infiltration	[184]
	Metformin treatment	Mice tMCAO model	Alleviated neutrophil infiltration by downregulating AMPK-dependent ICAM-1	[186]
	Subcutaneous injection of monoclonal anti-HMGB1 antibody	5XFAD mice AD model	Blocked HMGB1 activity *via* TLR4	[197]
	Resveratrol treatment	AD patients	Inhibited MMP-9	[201]
	Blocking LFA-1 integrin	3xTg mice AD model	Prevented neutrophil adhesion and extravasation	[206]
	Activating RAGE/TLR4 signaling	Rat primary hippocampal neuron cultures	Inhibited NETs formation by suppressing HMGB1	[207]

**Abbreviations:** MIF, macrophage migration inhibitory factor;4-IPP, 4-iodo-6-phenylpyrimidine; tMCAO, transient middle cerebral artery occlusion; MCA, middle cerebral artery; HOCL, hypochlorite; MPO, myeloperoxidase; cSLNs, cationic solid lipid nanoparticles; NE, neutrophil elastase; NET, neutrophil extracellular trap; AVM, avermectin; H_2_S, hydrogen sulfide; PTEN, phosphatase and tensin homolog deleted on chromosome 10; nNIF, neonatal NET-inhibitory factor; PAD4, peptidylarginine deiminase 4; RAGE Receptor for Advanced Glycation End products; BB-FCF, Brilliant blue FCF dye; Panx1, pannexin1; TLR4, Toll-like receptor 4; ABAH, 4-aminobenzoic acid hydrazide; Hsp70, heat shock protein 70; AD, Alzheimer’s disease; MMP, Matrix metalloproteinase; HMGB1, high mobility group box 1 protein; tPA, tissue-type plasminogen activator; IFN, interferon; ICS II, icariside II; TIMP, tissue inhibitor of metalloproteinase.

## LIST OF ABBREVIATIONS

AD: Alzheimer's Disease
AF: Atrial Fibrillation
AIS: Acute Ischemic Stroke
ATP: Adenosine Triphosphate
BBB: Brain-blood Barrier
CIRI: Cerebral Ischemic Reperfusion Injury
CNS: Central Nervous System
MI: Myocardial Infarction
MMPs: Matrix Metalloproteinases
MPO: Myeloperoxidase
MS: Multiple Sclerosis
NE: Neutrophil Elastase
NETs: Neutrophil Extracellular Traps
PSD: Post-stroke Depression
ROS: Reactive Oxygen Species
TF: Tissue Factor
TFPI: Tissue Factor Pathway Inhibitors

## References

[1] Lin H.W., Lee R.C., Lee M.H.H., Wu C.Y.C., Couto e Silva A., Possoit H.E., Hsieh T-H., Minagar A. (2018). Cerebral ischemia and neuroregeneration.. Neural Regen. Res..

[2] Kuriakose D., Xiao Z. (2020). Pathophysiology and Treatment of Stroke: Present Status and Future Perspectives.. Int. J. Mol. Sci..

[3] Villain N., Dubois B. (2019). Alzheimer’s Disease Including Focal Presentations.. Semin. Neurol..

[4] Hurford R., Sekhar A., Hughes T.A.T., Muir K.W. (2020). Diagnosis and management of acute ischaemic stroke.. Pract. Neurol..

[5] Kalogeris T., Baines C.P., Krenz M., Korthuis R.J. (2012). Cell biology of ischemia/reperfusion injury.. Int. Rev. Cell Mol. Biol..

[6] Jian Z., Liu R., Zhu X., Smerin D., Zhong Y., Gu L., Fang W., Xiong X. (2019). The Involvement and therapy target of immune cells after ischemic stroke.. Front. Immunol..

[7] Brinkmann V., Reichard U., Goosmann C., Fauler B., Uhlemann Y., Weiss D.S., Weinrauch Y., Zychlinsky A. (2004). Neutrophil extracellular traps kill bacteria.. Science.

[8] Klopf J., Brostjan C., Eilenberg W., Neumayer C. (2021). Neutrophil extracellular traps and their implications in cardiovascular and inflammatory disease.. Int. J. Mol. Sci..

[9] Mutua V., Gershwin L.J. (2021). A review of neutrophil extracellular traps (NETs) in disease: Potential anti-NETs therapeutics.. Clin. Rev. Allergy Immunol..

[10] Vorobjeva N.V., Chernyak B.V. (2020). NETosis: Molecular mechanisms, role in physiology and pathology.. Biochemistry (Mosc.).

[11] Guo Y., Zeng H., Gao C. (2021). The role of neutrophil extracellular traps in central nervous system diseases and prospects for clinical application.. Oxid Med Cell Longev..

[12] Fuchs T.A., Brill A., Duerschmied D., Schatzberg D., Monestier M., Myers D.D., Wrobleski S.K., Wakefield T.W., Hartwig J.H., Wagner D.D. (2010). Extracellular DNA traps promote thrombosis.. Proc. Natl. Acad. Sci. USA.

[13] Thålin C., Hisada Y., Lundström S., Mackman N., Wallén H. (2019). Neutrophil extracellular traps.. Arterioscler. Thromb. Vasc. Biol..

[14] Hahn J., Schauer C., Czegley C., Kling L., Petru L., Schmid B., Weidner D., Reinwald C., Biermann M.H.C., Blunder S., Ernst J., Lesner A., Bäuerle T., Palmisano R., Christiansen S., Herrmann M., Bozec A., Gruber R., Schett G., Hoffmann M.H. (2019). Aggregated neutrophil extracellular traps resolve inflammation by proteolysis of cytokines and chemokines and protection from antiproteases.. FASEB J..

[15] Bonaventura A., Vecchié A., Abbate A., Montecucco F. (2020). Neutrophil extracellular traps and cardiovascular diseases: An update.. Cells.

[16] Manda-Handzlik A., Demkow U. (2019). The brain entangled: The contribution of neutrophil extracellular traps to the diseases of the central nervous system.. Cells.

[17] Allen C., Thornton P., Denes A., McColl B.W., Pierozynski A., Monestier M., Pinteaux E., Rothwell N.J., Allan S.M. (2012). Neutrophil cerebrovascular transmigration triggers rapid neurotoxicity through release of proteases associated with decondensed DNA.. J. Immunol..

[18] Kim S.W., Lee H., Lee H.K., Kim I.D., Lee J.K. (2019). Neutrophil extracellular trap induced by HMGB1 exacerbates damages in the ischemic brain.. Acta Neuropathol. Commun..

[19] Othman A., Sekheri M., Filep J.G. (2022). Roles of neutrophil granule proteins in orchestrating inflammation and immunity.. FEBS J..

[20] Domínguez-Díaz C., Varela-Trinidad G.U., Muñoz-Sánchez G., Solórzano-Castanedo K., Avila-Arrezola K.E., Iñiguez-Gutiérrez L., Delgado-Rizo V., Fafutis-Morris M. (2021). To trap a pathogen: Neutrophil extracellular traps and their role in mucosal epithelial and skin diseases.. Cells.

[21] Urban C.F., Ermert D., Schmid M., Abu-Abed U., Goosmann C., Nacken W., Brinkmann V., Jungblut P.R., Zychlinsky A. (2009). Neutrophil extracellular traps contain calprotectin, a cytosolic protein complex involved in host defense against Candida albicans.. PLoS Pathog..

[22] Rada B. (2019). Neutrophil extracellular traps.. Methods Mol. Biol..

[23] Vallés J., Santos M.T., Latorre A.M., Tembl J., Salom J., Nieves C., Lago A., Moscardó A. (2017). Neutrophil extracellular traps are increased in patients with acute ischemic stroke: prognostic significance.. Thromb. Haemost..

[24] He Y., Yang F.Y., Sun E.W. (2018). Neutrophil extracellular traps in autoimmune diseases.. Chin. Med. J. (Engl.).

[25] Carestia A., Kaufman T., Schattner M. (2016). Platelets: New bricks in the building of neutrophil extracellular traps.. Front. Immunol..

[26] Chen R., Zhang X., Gu L., Zhu H., Zhong Y., Ye Y., Xiong X., Jian Z. (2021). New insight into neutrophils: A potential therapeutic target for cerebral ischemia.. Front. Immunol..

[27] Rohrbach A.S., Hemmers S., Arandjelovic S., Corr M., Mowen K.A. (2012). PAD4 is not essential for disease in the K/BxN murine autoantibody-mediated model of arthritis.. Arthritis Res. Ther..

[28] Narasaraju T., Yang E., Samy R.P., Ng H.H., Poh W.P., Liew A.A., Phoon M.C., van Rooijen N., Chow V.T. (2011). Excessive neutrophils and neutrophil extracellular traps contribute to acute lung injury of influenza pneumonitis.. Am. J. Pathol..

[29] Ravindran M., Khan M.A., Palaniyar N. (2019). Neutrophil extracellular trap formation: Physiology, pathology, and pharmacology.. Biomolecules.

[30] van Dam L.S., Rabelink T.J., van Kooten C., Teng Y.K.O. (2019). Clinical implications of excessive neutrophil extracellular trap formation in renal autoimmune diseases.. Kidney Int. Rep..

[31] Douda D.N., Khan M.A., Grasemann H., Palaniyar N. (2015). SK3 channel and mitochondrial ROS mediate NADPH oxidase-independent NETosis induced by calcium influx.. Proc. Natl. Acad. Sci. USA.

[32] Njeim R., Azar W.S., Fares A.H., Azar S.T., Kfoury Kassouf H., Eid A.A. (2020). NETosis contributes to the pathogenesis of diabetes and its complications.. J. Mol. Endocrinol..

[33] Deng J., Zhao F., Zhang Y., Zhou Y., Xu X., Zhang X., Zhao Y. (2020). Neutrophil extracellular traps increased by hyperglycemia exacerbate ischemic brain damage.. Neurosci. Lett..

[34] Dziedzic A., Saluk-Bijak J., Miller E., Bijak M. (2020). Metformin as a potential agent in the treatment of multiple sclerosis.. Int. J. Mol. Sci..

[35] Menegazzo L., Scattolini V., Cappellari R., Bonora B.M., Albiero M., Bortolozzi M., Romanato F., Ceolotto G., Vigili de Kreutzeberg S., Avogaro A., Fadini G.P. (2018). The antidiabetic drug metformin blunts NETosis in vitro and reduces circulating NETosis biomarkers in vivo.. Acta Diabetol..

[36] Fousert E., Toes R., Desai J. (2020). Neutrophil extracellular traps (NETs) take the central stage in driving autoimmune responses.. Cells.

[37] Lee K.H., Kronbichler A., Park D.D.Y., Park Y., Moon H., Kim H., Choi J.H., Choi Y., Shim S., Lyu I.S., Yun B.H., Han Y., Lee D., Lee S.Y., Yoo B.H., Lee K.H., Kim T.L., Kim H., Shim J.S., Nam W., So H., Choi S., Lee S., Shin J.I. (2017). Neutrophil extracellular traps (NETs) in autoimmune diseases: A comprehensive review.. Autoimmun. Rev..

[38] Döring Y., Soehnlein O., Weber C. (2017). Neutrophil extracellular traps in atherosclerosis and atherothrombosis.. Circ. Res..

[39] Qi H., Yang S., Zhang L. (2017). Neutrophil extracellular traps and endothelial dysfunction in atherosclerosis and thrombosis.. Front. Immunol..

[40] Kessenbrock K., Krumbholz M., Schönermarck U., Back W., Gross W.L., Werb Z., Gröne H.J., Brinkmann V., Jenne D.E. (2009). Netting neutrophils in autoimmune small-vessel vasculitis.. Nat. Med..

[41] Kretzschmar G.C., Bumiller-Bini V., Gasparetto Filho M.A., Zonta Y.R., Yu K.S.T., de Souza R.L.R., Dias-Melicio L.A., Boldt A.B.W. (2021). Neutrophil extracellular traps: A perspective of neuroinflammation and complement activation in Alzheimer’s disease.. Front. Mol. Biosci..

[42] Lim S., Kim T.J., Kim Y.J., Kim C., Ko S.B., Kim B.S. (2021). Senolytic therapy for cerebral ischemia-reperfusion injury.. Int. J. Mol. Sci..

[43] Otxoa-de-Amezaga A., Gallizioli M., Pedragosa J., Justicia C., Miró-Mur F., Salas-Perdomo A., Díaz-Marugan L., Gunzer M., Planas A.M. (2019). Location of neutrophils in different compartments of the damaged mouse brain after severe ischemia/reperfusion.. Stroke.

[44] Goktay A.Y., Senturk C. (2016). Endovascular treatment of thrombosis and embolism.. Adv. Exp. Med. Biol..

[45] Laridan E., Denorme F., Desender L., François O., Andersson T., Deckmyn H., Vanhoorelbeke K., De Meyer S.F. (2017). Neutrophil extracellular traps in ischemic stroke thrombi.. Ann. Neurol..

[46] Kim J.E., Yoo H.J., Gu J.Y., Kim H.K. (2016). Histones induce the procoagulant phenotype of endothelial cells through tissue factor up-regulation and thrombomodulin down-regulation.. PLoS One.

[47] Wu X., Zeng H., Cai L., Chen G. (2021). Role of the extracellular traps in central nervous system.. Front. Immunol..

[48] Vorobjeva N.V., Pinegin B.V. (2014). Neutrophil Extracellular Traps: Mechanisms of formation and role in health and disease.. Biochemistry (Mosc.).

[49] Zhou P., Li T., Jin J., Liu Y., Li B., Sun Q., Tian J., Zhao H., Liu Z., Ma S., Zhang S., Novakovic V.A., Shi J., Hu S. (2020). Interactions between neutrophil extracellular traps and activated platelets enhance procoagulant activity in acute stroke patients with ICA occlusion.. EBioMedicine.

[50] von Brühl M.L., Stark K., Steinhart A., Chandraratne S., Konrad I., Lorenz M., Khandoga A., Tirniceriu A., Coletti R., Köllnberger M., Byrne R.A., Laitinen I., Walch A., Brill A., Pfeiler S., Manukyan D., Braun S., Lange P., Riegger J., Ware J., Eckart A., Haidari S., Rudelius M., Schulz C., Echtler K., Brinkmann V., Schwaiger M., Preissner K.T., Wagner D.D., Mackman N., Engelmann B., Massberg S. (2012). Monocytes, neutrophils, and platelets cooperate to initiate and propagate venous thrombosis in mice in vivo.. J. Exp. Med..

[51] Varjú I., Kolev K. (2019). Networks that stop the flow: A fresh look at fibrin and neutrophil extracellular traps.. Thromb. Res..

[52] Schattner M. (2019). Platelet TLR4 at the crossroads of thrombosis and the innate immune response.. J. Leukoc. Biol..

[53] Fuchs T.A., Bhandari A.A., Wagner D.D. (2011). Histones induce rapid and profound thrombocytopenia in mice.. Blood.

[54] Peña-Martínez C., Durán-Laforet V., García-Culebras A., Cuartero M.I., Moro M.Á., Lizasoain I. (2022). Neutrophil extracellular trap targeting protects against ischemic damage after fibrin-rich thrombotic stroke despite non-reperfusion.. Front. Immunol..

[55] Sambrano G.R., Huang W., Faruqi T., Mahrus S., Craik C., Coughlin S.R. (2000). Cathepsin G activates protease-activated receptor-4 in human platelets.. J. Biol. Chem..

[56] Mihara K., Ramachandran R., Renaux B., Saifeddine M., Hollenberg M.D. (2013). Neutrophil elastase and proteinase-3 trigger G protein-biased signaling through proteinase-activated receptor-1 (PAR1).. J. Biol. Chem..

[57] Li C., Xing Y., Zhang Y., Hua Y., Hu J., Bai Y. (2022). Neutrophil extracellular traps exacerbate ischemic brain damage.. Mol. Neurobiol..

[58] Tadie J.M., Bae H.B., Jiang S., Park D.W., Bell C.P., Yang H., Pittet J.F., Tracey K., Thannickal V.J., Abraham E., Zmijewski J.W. (2013). HMGB1 promotes neutrophil extracellular trap formation through interactions with Toll-like receptor 4.. Am. J. Physiol. Lung Cell. Mol. Physiol..

[59] Kim S.W., Lee J.K. (2020). Role of HMGB1 in the Interplay between NETosis and thrombosis in ischemic stroke: A review.. Cells.

[60] Martinod K., Wagner D.D. (2014). Thrombosis: tangled up in NETs.. Blood.

[61] Seners P., Turc G., Maïer B., Mas J.L., Oppenheim C., Baron J.C. (2016). Incidence and predictors of early recanalization after intravenous thrombolysis.. Stroke.

[62] Farrera C., Fadeel B. (2013). Macrophage clearance of neutrophil extracellular traps is a silent process.. J. Immunol..

[63] Zhang S., Cao Y., Du J., Liu H., Chen X., Li M., Xiang M., Wang C., Wu X., Liu L., Wang C., Wu Y., Li Z., Fang S., Shi J., Wang L. (2021). Neutrophil extracellular traps contribute to tissue plasminogen activator resistance in acute ischemic stroke.. FASEB J..

[64] Ducroux C., Di Meglio L., Loyau S., Delbosc S., Boisseau W., Deschildre C., Ben Maacha M., Blanc R., Redjem H., Ciccio G., Smajda S., Fahed R., Michel J.B., Piotin M., Salomon L., Mazighi M., Ho-Tin-Noe B., Desilles J.P. (2018). Thrombus neutrophil extracellular traps content impair tPA-induced thrombolysis in acute ischemic stroke.. Stroke.

[65] Cahilog Z., Zhao H., Wu L., Alam A., Eguchi S., Weng H., Ma D. (2020). The role of neutrophil NETosis in organ injury: Novel inflammatory cell death mechanisms.. Inflammation.

[66] Liu K., Mori S., Takahashi H.K., Tomono Y., Wake H., Kanke T., Sato Y., Hiraga N., Adachi N., Yoshino T., Nishibori M. (2007). Anti‐high mobility group box 1 monoclonal antibody ameliorates brain infarction induced by transient ischemia in rats.. FASEB J..

[67] Denorme F., Portier I., Rustad J.L., Cody M.J., de Araujo C.V., Hoki C., Alexander M.D., Grandhi R., Dyer M.R., Neal M.D., Majersik J.J., Yost C.C., Campbell R.A. (2022). Neutrophil extracellular traps regulate ischemic stroke brain injury.. J. Clin. Invest..

[68] Vogel S., Bodenstein R., Chen Q., Feil S., Feil R., Rheinlaender J., Schäffer T.E., Bohn E., Frick J.S., Borst O., Münzer P., Walker B., Markel J., Csanyi G., Pagano P.J., Loughran P., Jessup M.E., Watkins S.C., Bullock G.C., Sperry J.L., Zuckerbraun B.S., Billiar T.R., Lotze M.T., Gawaz M., Neal M.D. (2015). Platelet-derived HMGB1 is a critical mediator of thrombosis.. J. Clin. Invest..

[69] El-Benna J., Hurtado-Nedelec M., Marzaioli V., Marie J.C., Gougerot-Pocidalo M.A., Dang P.M.C. (2016). Priming of the neutrophil respiratory burst: role in host defense and inflammation.. Immunol. Rev..

[70] Wang J., Jiang Y., Zeng D., Zhou W., Hong X. (2020). Prognostic value of plasma HMGB1 in ischemic stroke patients with cerebral ischemia-reperfusion injury after intravenous thrombolysis.. J. Stroke Cerebrovasc. Dis..

[71] Chen Y., Zhang H., Hu X., Cai W., Ni W., Zhou K. (2022). Role of NETosis in central nervous system injury.. Oxid. Med. Cell. Longev..

[72] Jorch S.K., Kubes P. (2017). An emerging role for neutrophil extracellular traps in noninfectious disease.. Nat. Med..

[73] Chen S., Chen H., Du Q., Shen J. (2020). Targeting myeloperoxidase (MPO) mediated oxidative stress and inflammation for reducing brain ischemia injury: Potential application of natural compounds.. Front. Physiol..

[74] Furtmüller P.G., Obinger C., Hsuanyu Y., Dunford H.B. (2000). Mechanism of reaction of myeloperoxidase with hydrogen peroxide and chloride ion.. Eur. J. Biochem..

[75] Yap Y.W., Whiteman M., Cheung N.S. (2007). Chlorinative stress: An under appreciated mediator of neurodegeneration?. Cell. Signal..

[76] Weiss S.J., Klein R., Slivka A., Wei M. (1982). Chlorination of taurine by human neutrophils. Evidence for hypochlorous acid generation.. J. Clin. Invest..

[77] Schraufstätter I.U., Browne K., Harris A., Hyslop P.A., Jackson J.H., Quehenberger O., Cochrane C.G. (1990). Mechanisms of hypochlorite injury of target cells.. J. Clin. Invest..

[78] Prütz W.A. (1996). Hypochlorous acid interactions with thiols, nucleotides, DNA, and other biological substrates.. Arch. Biochem. Biophys..

[79] Panasenko O.M. (1997). The mechanism of the hypochlorite-induced lipid peroxidation.. Biofactors.

[80] Hawkins C.L., Pattison D.I., Davies M.J. (2003). Hypochlorite-induced oxidation of amino acids, peptides and proteins.. Amino Acids.

[81] Pattison D.I., Hawkins C.L., Davies M.J. (2003). Hypochlorous acid-mediated oxidation of lipid components and antioxidants present in low-density lipoproteins: absolute rate constants, product analysis, and computational modeling.. Chem. Res. Toxicol..

[82] Thai T., Zhong F., Dang L., Chan E., Ku J., Malle E., Geczy C.L., Keaney J.F., Thomas S.R. (2021). Endothelial-transcytosed myeloperoxidase activates endothelial nitric oxide synthase via a phospholipase C-dependent calcium signaling pathway.. Free Radic. Biol. Med..

[83] Guan W., Zhao Y., Xu C. (2011). A Combined treatment with taurine and intra-arterial thrombolysis in an embolic model of stroke in rats: Increased neuroprotective efficacy and extended therapeutic time window.. Transl. Stroke Res..

[84] Ayloo S., Gu C. (2019). Transcytosis at the blood-brain barrier.. Curr. Opin. Neurobiol..

[85] Yang C., Hawkins K.E., Doré S., Candelario-Jalil E. (2019). Neuroinflammatory mechanisms of blood-brain barrier damage in ischemic stroke.. Am. J. Physiol. Cell Physiol..

[86] Strecker J.K., Schmidt A., Schäbitz W.R., Minnerup J. (2017). Neutrophil granulocytes in cerebral ischemia - Evolution from killers to key players.. Neurochem. Int..

[87] Jian-gang M., Gang Y. (2021). Advances in the study of neutrophil extracellular traps in ischemic stroke.. Hainan Med J..

[88] Lee C.Z., Xue Z., Zhu Y., Yang G.Y., Young W.L. (2007). Matrix metalloproteinase-9 inhibition attenuates vascular endothelial growth factor-induced intracerebral hemorrhage.. Stroke.

[89] Nakamura K., Nakayama H., Sasaki S., Takahashi K., Iwabuchi K. (2022). Mycobacterium avium-intracellulare complex promote release of pro-inflammatory enzymes matrix metalloproteinases by inducing neutrophil extracellular trap formation.. Sci. Rep..

[90] Yabluchanskiy A., Ma Y., Iyer R.P., Hall M.E., Lindsey M.L. (2013). Matrix metalloproteinase-9: Many shades of function in cardiovascular disease.. Physiology (Bethesda).

[91] Ballabh P., Braun A., Nedergaard M. (2004). The blood-brain barrier: an overview.. Neurobiol. Dis..

[92] Li Y., Zhong W., Jiang Z., Tang X. (2019). New progress in the approaches for blood-brain barrier protection in acute ischemic stroke.. Brain Res. Bull..

[93] Sifat A.E., Vaidya B., Abbruscato T.J. (2017). Blood-brain barrier protection as a therapeutic strategy for acute ischemic stroke.. AAPS J..

[94] Turner R.J., Sharp F.R. (2016). Implications of MMP9 for blood brain barrier disruption and hemorrhagic transformation following ischemic stroke.. Front. Cell. Neurosci..

[95] Kang L., Yu H., Yang X., Zhu Y., Bai X., Wang R., Cao Y., Xu H., Luo H., Lu L., Shi M.J., Tian Y., Fan W., Zhao B.Q. (2020). Neutrophil extracellular traps released by neutrophils impair revascularization and vascular remodeling after stroke.. Nat. Commun..

[96] Santos-Lima B., Pietronigro E.C., Terrabuio E., Zenaro E., Constantin G. (2022). The role of neutrophils in the dysfunction of central nervous system barriers.. Front. Aging Neurosci..

[97] Saffarzadeh M., Juenemann C., Queisser M.A., Lochnit G., Barreto G., Galuska S.P., Lohmeyer J., Preissner K.T. (2012). Neutrophil extracellular traps directly induce epithelial and endothelial cell death: a predominant role of histones.. PLoS One.

[98] Cuartero M.I., Ballesteros I., Moraga A., Nombela F., Vivancos J., Hamilton J.A., Corbí Á.L., Lizasoain I., Moro M.A. (2013). N2 neutrophils, novel players in brain inflammation after stroke: modulation by the PPARγ agonist rosiglitazone.. Stroke.

[99] Cai W., Liu S., Hu M., Huang F., Zhu Q., Qiu W., Hu X., Colello J., Zheng S.G., Lu Z. (2020). Functional dynamics of neutrophils after ischemic stroke.. Transl. Stroke Res..

[100] Gou X., Ying J., Yue Y., Qiu X., Hu P., Qu Y., Li J., Mu D. (2020). The roles of high mobility group box 1 in cerebral ischemic injury.. Front. Cell. Neurosci..

[101] Scaffidi P., Misteli T., Bianchi M.E. (2002). Release of chromatin protein HMGB1 by necrotic cells triggers inflammation.. Nature.

[102] Xie W., Zhu T., Dong X., Nan F., Meng X., Zhou P., Sun G., Sun X. (2019). HMGB1-triggered inflammation inhibition of notoginseng leaf triterpenes against cerebral ischemia and reperfusion injury via MAPK and NF-κB signaling pathways.. Biomolecules.

[103] Lok K.Z., Basta M., Manzanero S., Arumugam T.V. (2015). Intravenous immunoglobulin (IVIg) dampens neuronal toll-like receptor-mediated responses in ischemia.. J. Neuroinflammation.

[104] Yu G., Liang Y., Zheng S., Zhang H. (2018). Inhibition of myeloperoxidase by N-acetyl lysyltyrosylcysteine amide reduces oxidative stress-mediated inflammation, neuronal damage, and neural stem cell injury in a murine model of stroke.. J. Pharmacol. Exp. Ther..

[105] Virani S.S., Alonso A., Aparicio H.J., Benjamin E.J., Bittencourt M.S., Callaway C.W., Carson A.P., Chamberlain A.M., Cheng S., Delling F.N., Elkind M.S.V., Evenson K.R., Ferguson J.F., Gupta D.K., Khan S.S., Kissela B.M., Knutson K.L., Lee C.D., Lewis T.T., Liu J., Loop M.S., Lutsey P.L., Ma J., Mackey J., Martin S.S., Matchar D.B., Mussolino M.E., Navaneethan S.D., Perak A.M., Roth G.A., Samad Z., Satou G.M., Schroeder E.B., Shah S.H., Shay C.M., Stokes A., VanWagner L.B., Wang N.Y., Tsao C.W. (2021). Heart disease and stroke statistics—2021 update.. Circulation.

[106] Qiu Y., Zhang C., Chen A., Wang H., Zhou Y., Li Y., Hu B. (2021). Immune cells in the BBB disruption after acute ischemic stroke: targets for immune therapy?. Front. Immunol..

[107] Powers W.J., Rabinstein A.A., Ackerson T., Adeoye O.M., Bambakidis N.C., Becker K., Biller J., Brown M., Demaerschalk B.M., Hoh B., Jauch E.C., Kidwell C.S., Leslie-Mazwi T.M., Ovbiagele B., Scott P.A., Sheth K.N., Southerland A.M., Summers D.V., Tirschwell D.L. (2019). Guidelines for the early management of patients with acute ischemic stroke: 2019 update to the 2018 guidelines for the early management of acute ischemic stroke: a guideline for healthcare professionals from the american heart association/american stroke association.. Stroke.

[108] Turc G., Bhogal P., Fischer U., Khatri P., Lobotesis K., Mazighi M., Schellinger P.D., Toni D., de Vries J., White P., Fiehler J. (2019). European stroke organisation (ESO)- european society for minimally invasive neurological therapy (ESMINT) guidelines on mechanical thrombectomy in acute ischemic stroke.. J. Neurointerv. Surg..

[109] Menon B.K., Al-Ajlan F.S., Najm M., Puig J., Castellanos M., Dowlatshahi D., Calleja A., Sohn S.I., Ahn S.H., Poppe A., Mikulik R., Asdaghi N., Field T.S., Jin A., Asil T., Boulanger J.M., Smith E.E., Coutts S.B., Barber P.A., Bal S., Subramanian S., Mishra S., Trivedi A., Dey S., Eesa M., Sajobi T., Goyal M., Hill M.D., Demchuk A.M. (2018). Association of clinical, imaging, and thrombus characteristics with recanalization of visible intracranial occlusion in patients with acute ischemic stroke.. JAMA.

[110] Baron J.C. (2019). Author Correction: Protecting the ischaemic penumbra as an adjunct to thrombectomy for acute stroke.. Nat. Rev. Neurol..

[111] Su X.T., Wang L., Ma S.M., Cao Y., Yang N.N., Lin L.L., Fisher M., Yang J.W., Liu C.Z. (2020). Mechanisms of acupuncture in the regulation of oxidative stress in treating ischemic stroke.. Oxid. Med. Cell. Longev..

[112] Kahles T., Brandes R.P. (2012). NADPH oxidases as therapeutic targets in ischemic stroke.. Cell. Mol. Life Sci..

[113] Chen H., Yoshioka H., Kim G.S., Jung J.E., Okami N., Sakata H., Maier C.M., Narasimhan P., Goeders C.E., Chan P.H. (2011). Oxidative stress in ischemic brain damage: mechanisms of cell death and potential molecular targets for neuroprotection.. Antioxid. Redox Signal..

[114] Nathan C., Ding A. (2010). SnapShot: Reactive oxygen intermediates (ROI).. Cell.

[115] Allen C.L., Bayraktutan U. (2009). Oxidative stress and its role in the pathogenesis of ischaemic stroke.. Int. J. Stroke.

[116] Kahles T., Luedike P., Endres M., Galla H.J., Steinmetz H., Busse R., Neumann-Haefelin T., Brandes R.P. (2007). NADPH oxidase plays a central role in blood-brain barrier damage in experimental stroke.. Stroke.

[117] Casas A.I., Geuss E., Kleikers P.W.M., Mencl S., Herrmann A.M., Buendia I., Egea J., Meuth S.G., Lopez M.G., Kleinschnitz C., Schmidt H.H.H.W. (2017). NOX4-dependent neuronal autotoxicity and BBB breakdown explain the superior sensitivity of the brain to ischemic damage.. Proc. Natl. Acad. Sci. USA.

[118] Canty T.G., Boyle E.M., Farr A., Morgan E.N., Verrier E.D., Pohlman T.H. (1999). Oxidative stress induces NF-kappaB nuclear translocation without degradation of IkappaBalpha.. Circulation.

[119] Kleinschnitz C., Grund H., Wingler K., Armitage M.E., Jones E., Mittal M., Barit D., Schwarz T., Geis C., Kraft P., Barthel K., Schuhmann M.K., Herrmann A.M., Meuth S.G., Stoll G., Meurer S., Schrewe A., Becker L., Gailus-Durner V., Fuchs H., Klopstock T., de Angelis M.H., Jandeleit-Dahm K., Shah A.M., Weissmann N., Schmidt H.H.H.W. (2010). Post-stroke inhibition of induced NADPH oxidase type 4 prevents oxidative stress and neurodegeneration.. PLoS Biol..

[120] Fuchs T.A., Abed U., Goosmann C., Hurwitz R., Schulze I., Wahn V., Weinrauch Y., Brinkmann V., Zychlinsky A. (2007). Novel cell death program leads to neutrophil extracellular traps.. J. Cell Biol..

[121] Lood C., Blanco L.P., Purmalek M.M., Carmona-Rivera C., De Ravin S.S., Smith C.K., Malech H.L., Ledbetter J.A., Elkon K.B., Kaplan M.J. (2016). Neutrophil extracellular traps enriched in oxidized mitochondrial DNA are interferogenic and contribute to lupus-like disease.. Nat. Med..

[122] Gaul D.S., Weber J., van Tits L.J., Sluka S., Pasterk L., Reiner M.F., Calatayud N., Lohmann C., Klingenberg R., Pahla J., Vdovenko D., Tanner F.C., Camici G.G., Eriksson U., Auwerx J., Mach F., Windecker S., Rodondi N., Lüscher T.F., Winnik S., Matter C.M. (2018). Loss of Sirt3 accelerates arterial thrombosis by increasing formation of neutrophil extracellular traps and plasma tissue factor activity.. Cardiovasc. Res..

[123] Vogelgesang A., Lange C., Blümke L., Laage G., Rümpel S., Langner S., Bröker B.M., Dressel A., Ruhnau J. (2017). Ischaemic stroke and the recanalization drug tissue plasminogen activator interfere with antibacterial phagocyte function.. J. Neuroinflammation.

[124] Papayannopoulos V., Metzler K.D., Hakkim A., Zychlinsky A. (2010). Neutrophil elastase and myeloperoxidase regulate the formation of neutrophil extracellular traps.. J. Cell Biol..

[125] Schindler L., Smyth L.C.D., Bernhagen J., Hampton M.B., Dickerhof N. (2021). Macrophage migration inhibitory factor (MIF) enhances hypochlorous acid production in phagocytic neutrophils.. Redox Biol..

[126] Hwang T.L., Aljuffali I.A., Hung C.F., Chen C.H., Fang J.Y. (2015). The impact of cationic solid lipid nanoparticles on human neutrophil activation and formation of neutrophil extracellular traps (NETs).. Chem. Biol. Interact..

[127] Hwang T.L., Sung C.T., Aljuffali I.A., Chang Y.T., Fang J.Y. (2014). Cationic surfactants in the form of nanoparticles and micelles elicit different human neutrophil responses: A toxicological study.. Colloids Surf. B Biointerfaces.

[128] Zheng S., Wang S., Zhang Q., Zhang Z., Xu S. (2020). Avermectin inhibits neutrophil extracellular traps release by activating PTEN demethylation to negatively regulate the PI3K-ERK pathway and reducing respiratory burst in carp.. J. Hazard. Mater..

[129] Yin K., Cui Y., Qu Y., Zhang J., Zhang H., Lin H. (2020). Hydrogen sulfide upregulates miR-16-5p targeting PiK3R1 and RAF1 to inhibit neutrophil extracellular trap formation in chickens.. Ecotoxicol. Environ. Saf..

[130] Leinweber J., Mizurini D.M., Francischetti I.M.B., Fleischer M., Hermann D.M., Kleinschnitz C., Langhauser F. (2021). Elastase inhibitor agaphelin protects from acute ischemic stroke in mice by reducing thrombosis, blood-brain barrier damage, and inflammation.. Brain Behav. Immun..

[131] De Meyer S.F., Denorme F., Langhauser F., Geuss E., Fluri F., Kleinschnitz C. (2016). Thromboinflammation in stroke brain damage.. Stroke.

[132] Burkard P., Vögtle T., Nieswandt B. (2020). Platelets in thrombo-inflammation: Concepts, mechanisms, and therapeutic strategies for ischemic stroke.. Hamostaseologie.

[133] Nieswandt B., Kleinschnitz C., Stoll G. (2011). Ischaemic stroke: a thrombo-inflammatory disease?. J. Physiol..

[134] Martinod K., Deppermann C. (2021). Immunothrombosis and thromboinflammation in host defense and disease.. Platelets.

[135] Maugeri N., Campana L., Gavina M., Covino C., De Metrio M., Panciroli C., Maiuri L., Maseri A., D’Angelo A., Bianchi M.E., Rovere-Querini P., Manfredi A.A. (2014). Activated platelets present high mobility group box 1 to neutrophils, inducing autophagy and promoting the extrusion of neutrophil extracellular traps.. J. Thromb. Haemost..

[136] Ma Y.H., Ma T., Wang C., Wang H., Chang D.Y., Chen M., Zhao M.H. (2016). High-mobility group box 1 potentiates antineutrophil cytoplasmic antibody-inducing neutrophil extracellular traps formation.. Arthritis Res. Ther..

[137] Karmakar M., Katsnelson M.A., Dubyak G.R., Pearlman E. (2016). Neutrophil P2X7 receptors mediate NLRP3 inflammasome-dependent IL-1β secretion in response to ATP.. Nat. Commun..

[138] Feng L., Chen Y., Ding R., Fu Z., Yang S., Deng X., Zeng J. (2015). P2X7R blockade prevents NLRP3 inflammasome activation and brain injury in a rat model of intracerebral hemorrhage: involvement of peroxynitrite.. J. Neuroinflammation.

[139] Chen Y., Yao Y., Sumi Y., Li A., To U.K., Elkhal A., Inoue Y., Woehrle T., Zhang Q., Hauser C., Junger W.G. (2010). Purinergic signaling: a fundamental mechanism in neutrophil activation.. Sci. Signal..

[140] Wang X., Chen D. (2018). Purinergic regulation of neutrophil function.. Front. Immunol..

[141] Sofoluwe A., Bacchetta M., Badaoui M., Kwak B.R., Chanson M. (2019). ATP amplifies NADPH-dependent and -independent neutrophil extracellular trap formation.. Sci. Rep..

[142] Brea D., Blanco M., Ramos-Cabrer P., Moldes O., Arias S., Pérez-Mato M., Leira R., Sobrino T., Castillo J. (2011). Toll-like receptors 2 and 4 in ischemic stroke: Outcome and therapeutic values.. J. Cereb. Blood Flow Metab..

[143] Durán-Laforet V., Peña-Martínez C., García-Culebras A., Cuartero M.I., Lo E.H., Moro M.Á., Lizasoain I. (2021). Role of TLR4 in neutrophil dynamics and functions: Contribution to stroke pathophysiology.. Front. Immunol..

[144] Waisberg M., Molina-Cruz A., Mizurini D.M., Gera N., Sousa B.C., Ma D., Leal A.C., Gomes T., Kotsyfakis M., Ribeiro J.M.C., Lukszo J., Reiter K., Porcella S.F., Oliveira C.J., Monteiro R.Q., Barillas-Mury C., Pierce S.K., Francischetti I.M.B. (2014). Plasmodium falciparum infection induces expression of a mosquito salivary protein (Agaphelin) that targets neutrophil function and inhibits thrombosis without impairing hemostasis.. PLoS Pathog..

[145] Ikegame Y., Yamashita K., Hayashi S., Yoshimura S., Nakashima S., Iwama T. (2010). Neutrophil elastase inhibitor prevents ischemic brain damage via reduction of vasogenic edema.. Hypertens. Res..

[146] Stowe A.M., Adair-Kirk T.L., Gonzales E.R., Perez R.S., Shah A.R., Park T.S., Gidday J.M. (2009). Neutrophil elastase and neurovascular injury following focal stroke and reperfusion.. Neurobiol. Dis..

[147] Forghani R., Kim H.J., Wojtkiewicz G.R., Bure L., Wu Y., Hayase M., Wei Y., Zheng Y., Moskowitz M.A., Chen J.W. (2015). Myeloperoxidase propagates damage and is a potential therapeutic target for subacute stroke.. J. Cereb. Blood Flow Metab..

[148] Forghani R., Wojtkiewicz G.R., Zhang Y., Seeburg D., Bautz B.R.M., Pulli B., Milewski A.R., Atkinson W.L., Iwamoto Y., Zhang E.R., Etzrodt M., Rodriguez E., Robbins C.S., Swirski F.K., Weissleder R., Chen J.W. (2012). Demyelinating diseases: myeloperoxidase as an imaging biomarker and therapeutic target.. Radiology.

[149] Yenari M.A., Liu J., Zheng Z., Vexler Z.S., Lee J.E., Giffard R.G. (2005). Antiapoptotic and anti-inflammatory mechanisms of heat-shock protein protection.. Ann. N. Y. Acad. Sci..

[150] Zheng Z., Kim J.Y., Ma H., Lee J.E., Yenari M.A. (2008). Anti-inflammatory effects of the 70 kDa heat shock protein in experimental stroke.. J. Cereb. Blood Flow Metab..

[151] Kim H.J., Wei Y., Wojtkiewicz G.R., Lee J.Y., Moskowitz M.A., Chen J.W. (2019). Reducing myeloperoxidase activity decreases inflammation and increases cellular protection in ischemic stroke.. J. Cereb. Blood Flow Metab..

[152] Yang J., Wu Z., Long Q., Huang J., Hong T., Liu W., Lin J. (2020). Insights into immunothrombosis: the interplay among neutrophil extracellular trap, von willebrand factor, and ADAMTS13.. Front. Immunol..

[153] Guo J., Wang J., Sun W., Liu X. (2022). The advances of post-stroke depression: 2021 update.. J. Neurol..

[154] Popa-Wagner A., Sandu R.E., Buga A.M., Uzoni A., Petcu E.B. (2015). Neuroinflammation and comorbidities are frequently ignored factors in CNS pathology.. Neural Regen. Res..

[155] Wen H., Weymann K.B., Wood L., Wang Q.M. (2018). Inflammatory signaling in post-stroke fatigue and depression.. Eur. Neurol..

[156] Swardfager W., Winer D.A., Herrmann N., Winer S., Lanctôt K.L. (2013). Interleukin-17 in post-stroke neurodegeneration.. Neurosci. Biobehav. Rev..

[157] Park H., Li Z., Yang X.O., Chang S.H., Nurieva R., Wang Y.H., Wang Y., Hood L., Zhu Z., Tian Q., Dong C. (2005). A distinct lineage of CD4 T cells regulates tissue inflammation by producing interleukin 17.. Nat. Immunol..

[158] Huang S.U.S., O’Sullivan K.M. (2022). The Expanding role of extracellular traps in inflammation and autoimmunity: The new players in casting dark webs.. Int. J. Mol. Sci..

[159] Mora-Ruíz M.D., Blanco-Favela F., Chávez Rueda A.K., Legorreta-Haquet M.V., Chávez-Sánchez L. (2019). Role of interleukin-17 in acute myocardial infarction.. Mol. Immunol..

[160] Liao Y.H., Xia N., Zhou S.F., Tang T.T., Yan X.X., Lv B.J., Nie S.F., Wang J., Iwakura Y., Xiao H., Yuan J., Jevallee H., Wei F., Shi G.P., Cheng X. (2012). Interleukin-17A contributes to myocardial ischemia/reperfusion injury by regulating cardiomyocyte apoptosis and neutrophil infiltration.. J. Am. Coll. Cardiol..

[161] Hu J., Zhou W., Zhou Z., Yang Q., Han J., Yan Y., Dong W. (2019). Predictive value of inflammatory indicators for post-stroke depression in patients with ischemic stroke.. Nan Fang Yi Ke Da Xue Xue Bao.

[162] Beck H., Plate K.H. (2009). Angiogenesis after cerebral ischemia.. Acta Neuropathol..

[163] Essig F., Kollikowski A.M., Pham M., Solymosi L., Stoll G., Haeusler K.G., Kraft P., Schuhmann M.K. (2020). Immunohistological analysis of neutrophils and neutrophil extracellular traps in human thrombemboli causing acute ischemic stroke.. Int. J. Mol. Sci..

[164] (1995). Tissue plasminogen activator for acute ischemic stroke.. N. Engl. J. Med..

[165] Wang R., Zhu Y., Liu Z., Chang L., Bai X., Kang L., Cao Y., Yang X., Yu H., Shi M.J., Hu Y., Fan W., Zhao B.Q. (2021). Neutrophil extracellular traps promote tPA-induced brain hemorrhage via cGAS in mice with stroke.. Blood.

[166] Neeli I., Khan S.N., Radic M. (2008). Histone deimination as a response to inflammatory stimuli in neutrophils.. J. Immunol..

[167] Luo Y., Arita K., Bhatia M., Knuckley B., Lee Y.H., Stallcup M.R., Sato M., Thompson P.R. (2006). Inhibitors and inactivators of protein arginine deiminase 4: functional and structural characterization.. Biochemistry.

[168] Hamam H.J., Palaniyar N. (2019). Post-Translational Modifications in NETosis and NETs-Mediated Diseases.. Biomolecules.

[169] Wang Y., Li M., Stadler S., Correll S., Li P., Wang D., Hayama R., Leonelli L., Han H., Grigoryev S.A., Allis C.D., Coonrod S.A. (2009). Histone hypercitrullination mediates chromatin decondensation and neutrophil extracellular trap formation.. J. Cell Biol..

[170] Li P., Li M., Lindberg M.R., Kennett M.J., Xiong N., Wang Y. (2010). PAD4 is essential for antibacterial innate immunity mediated by neutrophil extracellular traps.. J. Exp. Med..

[171] Bicker K.L., Thompson P.R. (2013). The protein arginine deiminases: Structure, function, inhibition, and disease.. Biopolymers.

[172] Li C., Li Q., Mei Q., Lu T. (2015). Pharmacological effects and pharmacokinetic properties of icariin, the major bioactive component in Herba Epimedii.. Life Sci..

[173] Zhang D., Zhang J., Fong C., Yao X., Yang M. (2012). Herba epimedii flavonoids suppress osteoclastic differentiation and bone resorption by inducing G2/M arrest and apoptosis.. Biochimie.

[174] Liu Y.Q., Yang Q.X., Cheng M.C., Xiao H.B. (2014). Synergistic inhibitory effect of Icariside II with Icaritin from Herba Epimedii on pre-osteoclastic RAW264.7 cell growth.. Phytomedicine.

[175] Liu M., Wang W., Gao J., Li F., Shi J., Gong Q. (2020). Icariside II attenuates cerebral ischemia/reperfusion-induced blood-brain barrier dysfunction in rats via regulating the balance of MMP9/TIMP1.. Acta Pharmacol. Sin..

[176] Liu S., Li X., Gao J., Liu Y., Shi J., Gong Q. (2018). Icariside II, a phosphodiesterase-5 inhibitor, attenuates beta-amyloid-induced cognitive deficits via BDNF/TrkB/CREB signaling.. Cell. Physiol. Biochem..

[177] Zheng Y., Deng Y., Gao J., Lv C., Lang L., Shi J., Yu C., Gong Q. (2020). Icariside II inhibits lipopolysaccharide-induced inflammation and amyloid production in rat astrocytes by regulating IKK/IκB/NF-κB/BACE1 signaling pathway.. Acta Pharmacol. Sin..

[178] Yost C.C., Schwertz H., Cody M.J., Wallace J.A., Campbell R.A., Vieira-de-Abreu A., Araujo C.V., Schubert S., Harris E.S., Rowley J.W., Rondina M.T., Fulcher J.M., Koening C.L., Weyrich A.S., Zimmerman G.A. (2016). Neonatal NET-inhibitory factor and related peptides inhibit neutrophil extracellular trap formation.. J. Clin. Invest..

[179] Kim S.W., Davaanyam D., Seol S.I., Lee H.K., Lee H., Lee J.K. (2020). Adenosine triphosphate accumulated following cerebral ischemia induces neutrophil extracellular trap formation.. Int. J. Mol. Sci..

[180] Oboudiyat C., Glazer H., Seifan A., Greer C., Isaacson R. (2013). Alzheimer’s Disease.. Semin. Neurol..

[181] Querfurth H.W., LaFerla F.M. (2010). Alzheimer’s disease.. N. Engl. J. Med..

[182] Qiu C., Kivipelto M., von Strauss E. (2009). Epidemiology of Alzheimer’s disease: occurrence, determinants, and strategies toward intervention.. Dialogues Clin. Neurosci..

[183] Hays C.C., Zlatar Z.Z., Wierenga C.E. (2016). The utility of cerebral blood flow as a biomarker of preclinical Alzheimer’s disease.. Cell. Mol. Neurobiol..

[184] Bracko O., Njiru B.N., Swallow M., Ali M., Haft-Javaherian M., Schaffer C.B. (2020). Increasing cerebral blood flow improves cognition into late stages in Alzheimer’s disease mice.. J. Cereb. Blood Flow Metab..

[185] Zenaro E., Pietronigro E., Bianca V.D., Piacentino G., Marongiu L., Budui S., Turano E., Rossi B., Angiari S., Dusi S., Montresor A., Carlucci T., Nanì S., Tosadori G., Calciano L., Catalucci D., Berton G., Bonetti B., Constantin G. (2015). Neutrophils promote Alzheimer’s disease-like pathology and cognitive decline via LFA-1 integrin.. Nat. Med..

[186] Liu Y., Tang G., Li Y., Wang Y., Chen X., Gu X., Zhang Z., Wang Y., Yang G.Y. (2014). Metformin attenuates blood-brain barrier disruption in mice following middle cerebral artery occlusion.. J. Neuroinflammation.

[187] Cruz Hernández J.C., Bracko O., Kersbergen C.J., Muse V., Haft-Javaherian M., Berg M., Park L., Vinarcsik L.K., Ivasyk I., Rivera D.A., Kang Y., Cortes-Canteli M., Peyrounette M., Doyeux V., Smith A., Zhou J., Otte G., Beverly J.D., Davenport E., Davit Y., Lin C.P., Strickland S., Iadecola C., Lorthois S., Nishimura N., Schaffer C.B. (2019). Neutrophil adhesion in brain capillaries reduces cortical blood flow and impairs memory function in Alzheimer’s disease mouse models.. Nat. Neurosci..

[188] Grammas P., Samany P.G., Thirumangalakudi L. (2006). Thrombin and inflammatory proteins are elevated in Alzheimer’s disease microvessels: Implications for disease pathogenesis.. J. Alzheimers Dis..

[189] Grammas P., Ovase R. (2001). Inflammatory factors are elevated in brain microvessels in Alzheimer’s disease.. Neurobiol. Aging.

[190] Keshari R.S., Jyoti A., Dubey M., Kothari N., Kohli M., Bogra J., Barthwal M.K., Dikshit M. (2012). Cytokines induced neutrophil extracellular traps formation: implication for the inflammatory disease condition.. PLoS One.

[191] Jarre A., Gowert N.S., Donner L., Münzer P., Klier M., Borst O., Schaller M., Lang F., Korth C., Elvers M. (2014). Pre-activated blood platelets and a pro-thrombotic phenotype in APP23 mice modeling Alzheimer’s disease.. Cell. Signal..

[192] Gowert N.S., Donner L., Chatterjee M., Eisele Y.S., Towhid S.T., Münzer P., Walker B., Ogorek I., Borst O., Grandoch M., Schaller M., Fischer J.W., Gawaz M., Weggen S., Lang F., Jucker M., Elvers M. (2014). Blood platelets in the progression of Alzheimer’s disease.. PLoS One.

[193] Ferrer-Raventós P., Beyer K. (2021). Alternative platelet activation pathways and their role in neurodegenerative diseases.. Neurobiol. Dis..

[194] Donner L., Feige T., Freiburg C., Toska L.M., Reichert A.S., Chatterjee M., Elvers M. (2021). Impact of amyloid-β on platelet mitochondrial function and platelet-mediated amyloid aggregation in Alzheimer’s disease.. Int. J. Mol. Sci..

[195] Paudel Y.N., Angelopoulou E., Piperi C., Othman I., Aamir K., Shaikh M.F. (2020). Impact of HMGB1, RAGE, and TLR4 in Alzheimer’s disease (AD): From risk factors to therapeutic targeting.. Cells.

[196] Kong Z.H., Chen X., Hua H.P., Liang L., Liu L.J. (2017). The oral pretreatment of glycyrrhizin prevents surgery-induced cognitive impairment in aged mice by reducing neuroinflammation and Alzheimer’s-related pathology via HMGB1 inhibition.. J. Mol. Neurosci..

[197] Fujita K., Motoki K., Tagawa K., Chen X., Hama H., Nakajima K., Homma H., Tamura T., Watanabe H., Katsuno M., Matsumi C., Kajikawa M., Saito T., Saido T., Sobue G., Miyawaki A., Okazawa H. (2016). HMGB1, a pathogenic molecule that induces neurite degeneration via TLR4-MARCKS, is a potential therapeutic target for Alzheimer’s disease.. Sci. Rep..

[198] Volkman R., Ben-Zur T., Kahana A., Garty B.Z., Offen D. (2019). Myeloperoxidase deficiency inhibits cognitive decline in the 5XFAD mouse model of Alzheimer’s disease.. Front. Neurosci..

[199] Zenaro E., Piacentino G., Constantin G. (2017). The blood-brain barrier in Alzheimer’s disease.. Neurobiol. Dis..

[200] Ishihara K., Yamaguchi Y., Uchino S., Furuhashi T., Yamada S., Kihara S., Mori K., Ogawa M. (2006). ICAM-1 signal transduction in cells stimulated with neutrophil elastase.. Dig. Dis. Sci..

[201] Moussa C., Hebron M., Huang X., Ahn J., Rissman R.A., Aisen P.S., Turner R.S. (2017). Resveratrol regulates neuro-inflammation and induces adaptive immunity in Alzheimer’s disease.. J. Neuroinflammation.

[202] Wang Y., Rosen H., Madtes D.K., Shao B., Martin T.R., Heinecke J.W., Fu X. (2007). Myeloperoxidase inactivates TIMP-1 by oxidizing its N-terminal cysteine residue: an oxidative mechanism for regulating proteolysis during inflammation.. J. Biol. Chem..

[203] Itoh Y., Nagase H. (1995). Preferential inactivation of tissue inhibitor of metalloproteinases-1 that is bound to the precursor of matrix metalloproteinase 9 (progelatinase B) by human neutrophil elastase.. J. Biol. Chem..

[204] Gilthorpe J.D., Oozeer F., Nash J., Calvo M., Bennett D.L.H., Lumsden A., Pini A. (2013). Extracellular histone H1 is neurotoxic and drives a pro-inflammatory response in microglia.. F1000 Res..

[205] Duce J.A., Smith D.P., Blake R.E., Crouch P.J., Li Q.X., Masters C.L., Trounce I.A. (2006). Linker histone H1 binds to disease associated amyloid-like fibrils.. J. Mol. Biol..

[206] Pietronigro E.C., Della Bianca V., Zenaro E., Constantin G. (2017). NETosis in Alzheimer’s Disease.. Front. Immunol..

[207] Nan K., Han Y., Fang Q., Huang C., Yu L., Ge W., Xiang F., Tao Y.X., Cao H., Li J. (2019). HMGB1 gene silencing inhibits neuroinflammation via down-regulation of NF-κB signaling in primary hippocampal neurons induced by Aβ25-35.. Int. Immunopharmacol..

[208] Powers W.J., Derdeyn C.P., Biller J., Coffey C.S., Hoh B.L., Jauch E.C., Johnston K.C., Johnston S.C., Khalessi A.A., Kidwell C.S., Meschia J.F., Ovbiagele B., Yavagal D.R. (2015). 2015 American heart association/american stroke association focused update of the 2013 guidelines for the early management of patients with acute ischemic stroke regarding endovascular treatment.. Stroke.

[209] Hollist M., Morgan L., Cabatbat R., Au K., Kirmani M.F., Kirmani B.F. (2021). Acute stroke management: Overview and recent updates.. Aging Dis..

[210] Lim H.H., Jeong I.H., An G.D., Woo K.S., Kim K.H., Kim J.M., Yun S.H., Park J.I., Cha J.K., Kim M.H., Han J.Y. (2020). Evaluation of neutrophil extracellular traps as the circulating marker for patients with acute coronary syndrome and acute ischemic stroke.. J. Clin. Lab. Anal..

[211] Wang Y., Liu M., Pu C. (2017). 2014 Chinese guidelines for secondary prevention of ischemic stroke and transient ischemic attack.. Int. J. Stroke.

[212] Shinohara Y., Yanagihara T., Abe K., Yoshimine T., Fujinaka T., Chuma T., Ochi F., Nagayama M., Ogawa A., Suzuki N., Katayama Y., Kimura A., Minematsu K., II II. (2011). Cerebral infarction/transient ischemic attack (TIA).. J. Stroke Cerebrovasc. Dis..

[213] Xu L., Gao Y., Hu M., Dong Y., Xu J., Zhang J., Lv P. (2022). Edaravone dexborneol protects cerebral ischemia reperfusion injury through activating Nrf2/HO‐1 signaling pathway in mice.. Fundam. Clin. Pharmacol..

[214] Huang Y., Zhang X., Zhang C., Xu W., Li W., Feng Z., Zhang X., Zhao K. (2022). Edaravone dexborneol downregulates neutrophil extracellular trap expression and ameliorates blood-brain barrier permeability in acute ischemic stroke.. Mediators Inflamm..

